# Cabozantinib sensitizes microsatellite stable colorectal cancer to immune checkpoint blockade by immune modulation in human immune system mouse models

**DOI:** 10.3389/fonc.2022.877635

**Published:** 2022-11-07

**Authors:** Julie Lang, Alexis D. Leal, Juan A. Marín-Jiménez, Sarah J. Hartman, Jeremy Shulman, Natalie M. Navarro, Matthew S. Lewis, Anna Capasso, Stacey M. Bagby, Bethlehem W. Yacob, Morgan MacBeth, Brian M. Freed, S. Gail Eckhardt, Kimberly Jordan, Patrick J. Blatchford, Roberta Pelanda, Christopher H. Lieu, Wells A. Messersmith, Todd M. Pitts

**Affiliations:** ^1^ Department of Immunology and Microbiology, University of Colorado Anschutz Medical Campus, Aurora, CO, United States; ^2^ Division of Medical Oncology, Department of Medicine, University of Colorado School of Medicine, Aurora, CO, United States; ^3^ Department of Medical Oncology, Catalan Institute of Oncology (ICO-L´Hospitalet), Barcelona, Spain; ^4^ Department of Oncology, Livestrong Cancer Institutes, Dell Medical School, University of Texas at Austin, Austin, TX, United States; ^5^ Division of Allergy and Clinical Immunology, School of Medicine, University of Colorado, Anschutz Medical Campus, Aurora, CO, United States; ^6^ Department of Biostatistics and Informatics, Colorado School of Public Health, University of Colorado Denver, Aurora, CO, United States

**Keywords:** colorectal cancer, immunotherapy, tumor immunology, tumor microenvironment, humanized mouse model, immune checkpoint blockade, nivolumab, cabozantinib

## Abstract

Immune checkpoint inhibitors have been found to be effective in metastatic MSI-high colorectal cancers (CRC), however, have no efficacy in microsatellite stable (MSS) cancers, which comprise the majority of mCRC cases. Cabozantinib is a small molecule multi-tyrosine kinase inhibitor that is FDA approved in advanced renal cell, medullary thyroid, and hepatocellular carcinoma. Using Human Immune System (HIS) mice, we tested the ability of cabozantinib to prime MSS-CRC tumors to enhance the potency of immune checkpoint inhibitor nivolumab. In four independent experiments, we implanted distinct MSS-CRC patient-derived xenografts (PDXs) into the flanks of humanized BALB/c-Rag2^null^Il2rγ^null^Sirpα^NOD^ (BRGS) mice that had been engrafted with human hematopoietic stem cells at birth. For each PDX, HIS-mice cohorts were treated with vehicle, nivolumab, cabozantinib, or the combination. In three out of the four models, the combination had a lower tumor growth rate compared to vehicle or nivolumab-treated groups. Furthermore, interrogation of the HIS in immune organs and tumors by flow cytometry revealed increased Granzyme B+, TNFα+ and IFNγ+ CD4+ T cells among the human tumor infiltrating leukocytes (TIL) that correlated with reduced tumor growth in the combination-treated HIS-mice. Notably, slower growth correlated with increased expression of the CD4+ T cell ligand, HLA-DR, on the tumor cells themselves. Finally, the cabozantinib/nivolumab combination was tested in comparison to cobimetinib/atezolizumab. Although both combinations showed tumor growth inhibition, cabozantinib/nivolumab had enhanced cytotoxic IFNγ and TNFα+ T cells. This pre-clinical *in vivo* data warrants testing the combination in clinical trials for patients with MSS-CRC.

## Introduction

Colorectal cancer (CRC) is the 3rd most common cancer diagnosed in the U.S. and metastatic CRC (mCRC) is the 2nd leading cause of cancer-related death annually (1). Despite substantial improvements in treatment over the past decade, mCRC remains incurable with a median overall survival (OS) of approximately 30 months with optimal combination chemotherapy and biological agents, including anti-VEGF and anti-EGFR antibody (Ab) therapies (2). Regorafenib and trifluridine/tipiracil are the most recently approved agents for the treatment of refractory mCRC, however, these agents provide only modest improvements in OS when compared to best supportive care (3, 4). For this reason, further research and new drug development is urgently needed for the treatment of patients in this setting.

Immunotherapies which target immune regulatory checkpoints such as PD-1, PD-L1 and CTLA-4 have become the newest treatment options available in the anti-cancer armamentarium. Inhibition of these immune checkpoints (either singly or in combination) has led to significant success and durable anti-tumor responses in multiple tumor types (5–8). Immune checkpoint blockade (ICB) has been demonstrated to have antitumor efficacy in patients with mismatch repair deficient (dMMR)/MSI-high CRC (9, 10). However, these agents have not shown efficacy in those with mismatch repair proficient (pMMR)/MSS-CRC, which represents the vast majority of mCRC (~95%) (11). Given the limited treatment options for pMMR/MSS-CRC following progression on standard of care chemotherapy, there is a dire need to address how to overcome the resistance of these tumors to immunotherapy.

Cabozantinib is an orally bioavailable, potent inhibitor of MET, RET, AXL, FLT-3, KIT, ROS1, TIE-2, TRKB, TYRO3 and VEGFR-1, -2 and -3 and currently FDA approved for treatment of metastatic medullary thyroid carcinoma, metastatic renal cell carcinoma and metastatic/unresectable hepatocellular carcinoma (12). Cabozantinib has been previously studied in CRC xenograft studies. Sun, et al. reported reduced tumor volume and intratumoral microvessel density, with decreased expression of angiogenesis related proteins VEGF-A and CD31 in an HT-29 CRC xenograft model following treatment with cabozantinib (13). Work from our group has previously demonstrated potent anti-tumor activity of cabozantinib on 20 distinct CRC PDXs, with 80% (16/20) exhibiting sensitivity, defined as ≤ 20% tumor growth of treated compared to control (14). Additionally, when the effect of cabozantinib was compared to that of regorafenib, cabozantinib demonstrated significantly greater activity in a subset of these CRC PDX models (15). These data suggest that inhibition of both MET and VEGFR2 may result in increased anti-tumor activity in CRC. These findings led to a phase II study of single agent cabozantinib in refractory mCRC (NCT03542877). Preliminary data showed promising anti-tumor efficacy with 34 of the 44 enrolled patients evaluable for response: 1 partial response (PR) and 31 stable disease (SD) with a disease control rate (DCR) at 6 weeks of 73% (16). All patients who had achieved 12 week or longer progression free survival (PFS) in this study were MSS and BRAF wild-type.

There have recently been multiple clinical trials in various tumor types (including urothelial malignancies and gastrointestinal malignancies) to investigate the safety and efficacy of these combinations (17–24). Among these, the phase III IMblaze370 study randomized patients with mCRC in the third line setting and beyond to treatment with either atezolizumab in combination with cobimetinib, single-agent atezolizumab or regorafenib, and unfortunately failed to meet its primary endpoint of improved OS (23). Alternatively, the Japanese REGONIVO study was a phase Ib, open-label, dose-escalation and dose-expansion study of the combination of regorafenib and nivolumab in patients with advanced or metastatic gastric or CRC who were refractory or intolerant to standard of care therapies (20). This study showed objective tumor responses in 40% (20/50) of patients including 9 with mCRC (36%), 8 of which were MSS. However, a dose escalation/expansion study that enrolled 52 patients with refractory metastatic pMMR CRC to REGONIVO was recently completed and final results recently reported modest activity of the combination, with 3 of 40 evaluable patients achieving a PR and 22 with SD, mPFS of 4.3 months and mOS of 11.1 months (25). Unfortunately, these studies did not translate to clinically significant improvements in PFS or OS. Several studies are ongoing to investigate the safety and tolerability of various immunotherapy combinations in pMMR/MSS mCRC.

Further research is needed to determine effective treatment strategies to augment the immune response in patients with pMMR/MSS mCRC. To test potential rational immunotherapy combinations in pre-clinical models, immune competent *in vivo* models are needed, such as PDX models that have been humanized, to provide the proper immune construct to illicit an immune response. Our group has developed Human Immune System “HIS” mouse models through injection of human hematopoietic stem cells (HSCs) into immunodeficient BALB/c-Rag2^null^Il2rγ^null^Sirpα^NOD^ (BRGS) recipients that are subsequently implanted with MSS-CRC PDX (26–28). Within a few months after engraftment, the HIS-BRGS mice develop human B and T lymphocytes, and to a lesser degree, human myeloid cells from the transplanted HSCs. Importantly, several groups have shown that human allograft PDX tumors can grow in these mice at rates equivalent to the non-humanized immunodeficient host (27, 29). Furthermore, the T cells in the HIS-mice are phenotypically exhausted, a feature that allows this growth (28). The most important feature of this model is that the tumor microenvironment (TME) is maintained in these tumors, with higher human immune infiltration into “hot” tumors and less into “cold” tumors (27, 28, 30, 31). Thus, one can study the efficacy of combination immunotherapy and its ability to alter the TME.

In this manuscript, we report our findings from four experiments aimed at investigating the anti-tumor efficacy and immunogenicity of the combination of cabozantinib and nivolumab (Ca/N) in HIS-MSS-CRC-BRGS PDX mouse models. Finally, we compare the anti-tumor immune response in the HIS-CRC-BRGS mice to a fifth CRC PDX following treatment with Ca/N to another cohort treated with cobimetinib and atezolizumab (Co/A), a distinct combination therapy targeting tyrosine kinases and the PD-L1 pathway that has failed in the clinic in an effort to determine if these combinations result in differences in immune responses *in vivo* that may potentially explain the failure of Co/A in clinical trials and if Ca/N may be a superior immunotherapy combination in this patient population.

## Materials and methods

### PDX generation

All animal work was performed with approval by the University of Colorado Anschutz Medical Campus IACUC. Patient-derived tumor samples were collected from consenting CRC patients at the University of Colorado Cancer Center with approval by the Colorado Multiple Institutional Review Board. These samples were used to generate PDX models as described previously (**Supplementary Methods**) (32). Female athymic nude mice (aged 4-8 weeks) were purchased from Envigo (Indianapolis, IN) and implanted subcutaneously on the hind flanks with tumors sized approximately 3 mm^3^. When tumors reached approximately 1500mm^3^, tumors were harvested and used to generate HIS-BRGS-PDX, as described below.

### HIS-BRGS mice studies

**Figure 1:**
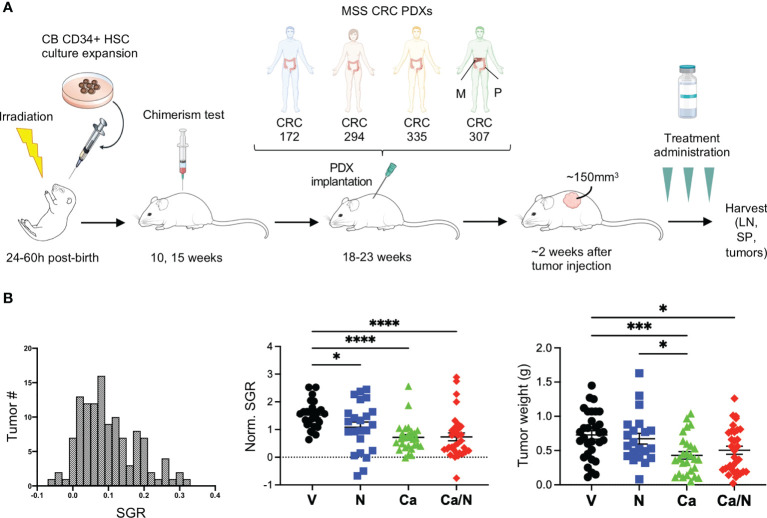
Reduced tumor growth in CRC MSS PDXs in HIS-BRGS mice treated with nivolumab, cabozantinib and their combination. **(A)** Experimental timeline for HIS-BRGS studies testing TKI/ICB treatments in five distinct CRC MSS PDX studies. Each PDX was tested in HIS-BRGS mice generated from a unique CB. **(B)** The distribution of number of tumors with specific growing rates (SGR) among all CRC PDX tumors in the HIS-BRGS studies (left panel); the normalized SGR among tumors in the nivolumab (N), cabozantinib (Ca) and their combination (Ca/N) treatment groups compared to untreated (V=vehicle) mice (middle panel); and CRC tumor weights at harvest in same treatment groups (right panel). P-values indicate ANOVA multiple comparisons with Welch’s correction: *p < 0.05, ***p < 0.001, ****p < 0.0001 or as indicated by number.

HIS-mice were generated from human CD34+ HSCs isolated from cord blood (CB) units, obtained as de-identified samples rejected from CB banking from Clinimmune and in compliance with University of Colorado Institutional Review Board (COMIRB #16-0541) as described previously (**Supplementary Methods**) (26, 33). Human B and T cell chimerism in the blood was confirmed at 10 and 15 weeks post engraftment using flow cytometry (28). CRC PDXs were implanted into both flanks of chimeric HIS-BRGS mice between 18-23 weeks of age (see experimental and PDX details in **Figure 1A**; **Supplemental Tables I**, **II**). This latter time point was chosen to ensure sufficient human T cells, as HIS mice are known to develop T cells 3-4 months after engraftment. For each PDX model, a cohort of HIS-BRGS-mice, prepared from the same CB (i.e. genetic donor), were allocated into four treatment groups consisting of 4-8 mice per cohort, depending on mouse number available. For details regarding the HIS-BRGS mouse model and experimental procedures for tumor immunotherapy studies, please refer to previous publications (27, 28, 34).

### Flow cytometry and analysis

Single cell suspensions of lymph nodes (LNs), spleens and tumors were stained and analyzed by flow cytometry as described previously (27, 28) using the Abs in **Supplemental Table III**. All samples were run for a set time and rate on either a Cyan 3-laser (CRC172, CRC294 and CRC335) or a Biorad Yeti 5-laser (CRC307M and CRC307P) flow cytometer. Data were analyzed using FlowJo software.

### Tumor growth measurement

In all the *in vivo* experiments, tumor growth rate has been measured using the specific growth rate (SGR), which was calculated using the following formula:


$$SGR=\frac{\ln(V_{2}/V_{1})}{t_{2}-t_{1}}$$

SGR quantifies the relative volume change (V_2_/V_1_) per time unit (t_2_ – t_1_). Thus, higher SGR values correspond to the fast-growing tumors whereas 0 and negative values represent non-growing and regressing tumors, respectively. SGR has demonstrated to be more accurate than the tumor volume doubling time for tumor growth estimation and a suitable parameter for statistical testing (35, 36).

### Statistics

Statistical analysis of immune measurements was performed using GraphPad Prism version 9.1.1 for MacOS, GraphPad Software, San Diego, California USA. Welch’s ANOVA test was used to compare means among three or more groups. For comparisons between two independent groups, unpaired t-test with Welch’s correction were performed. Simple linear regression analyses and reported R squared values were used to assess specific growth rate (SGR) (35) correlation with the different immune parameters.

## Results

### Cabozantinib alone and in combination with nivolumab reduces tumor growth of human PDXs in HIS-BRGS mouse models

We used the HIS-BRGS mouse pre-clinical model to evaluate the impact of Ca/N on growth and immune responses to human CRC tumors. To evaluate reproducibility and effects of tumor heterogeneity, we implanted four distinct CRC MSS PDXs into flanks of HIS-BRGS mice. For each PDX, HIS-BRGS mice were allocated into equivalent experimental groups (vehicle (V), nivolumab (N), cabozantinib (Ca) or combination (Ca/N)) based on the human CD45, CD3 and CD8 chimerism measured in the blood at 15 weeks (**Figure 1A** and **Supplemental Figure 1**). At the end of study, after removing mice that either died, did not grow tumors, or were low in chimerism, we ended up with the following group sizes for each PDX: CRC172 (V: 5, N: 4, Ca: 4, Ca/N: 5), CRC294 (V: 2, N: 5, Ca: 5, Ca/N: 4), CRC335: (V: 6, N: 4, Ca: 4, Ca/N: 6), CRC172M: (V: 5, N: 5, Ca: 6, Ca/N: 6).

To compare and quantify the tumor growth rate between the four PDX models, we calculated the SGR that measures the relative tumor growth per day of treatment (%/day) (36). The SGR across all PDXs showed a normal distribution (**Figure 1B**). Importantly, we observed reduced SGR and tumor weight at the end of treatment with both Ca and the combination of Ca/N (**Figure 1B**).

### Increased human T cells in spleens and TILs treated with Ca/N

**Figure 2:**
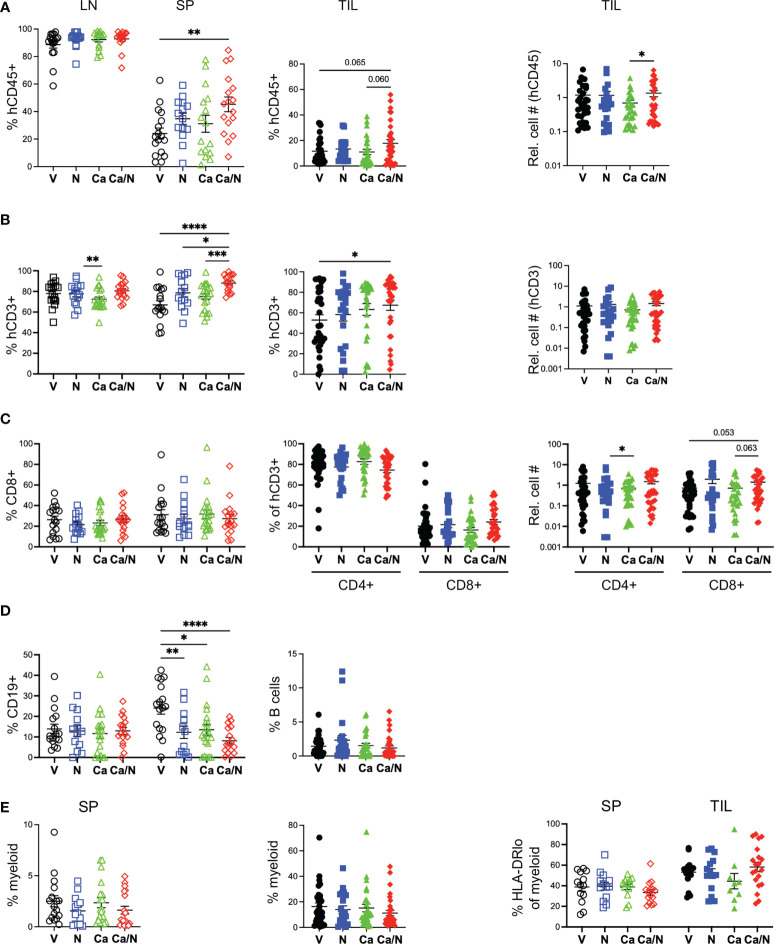
Increased human T cells in spleens and TILs of HIS-CRC-BRGS mice treated with combination cabozantinib and nivolumab. The HIS in LNs, spleens and tumors from HIS-CRC-BRGS mice were analyzed by flow cytometry for human immune subset frequencies (%) and cell numbers per g of tumor: **(A)** overall human (hCD45+) as a percentage of total immune cells (human + mouse CD45+), **(B)** T cells (CD3+), **(C)** CD4 and CD8+ T cells, **(D)** CD19+ B cells and **(E)** human myeloid cells as determined by markers CD11b, CD11c, CD14 or CD33. Right panel is frequency of myeloid cells with low HLA-DR class II expression. V=untreated, N=nivolumab alone, Ca=cabozantinib alone and Ca/N=combination cabozantinib and nivolumab. P-values indicate ANOVA multiple comparisons with Welch’s correction: *p < 0.05, **p < 0.01, ***p < 0.001, ****p < 0.0001 or as indicated by number.

At end of study, lymph organs and tumors were evaluated for the presence of mouse CD45 (mCD45^+^) and human CD45 (hCD45^+^) cells, as well as multiple human immune subsets (**Supplemental Figure 2A**). We observed a consistent increase in human chimerism in the spleens, but not the LNs, of the CRC-bearing HIS-BRGS mice receiving the combination treatment (**Figure 2A**). In the tumors, this increase in the frequency of hCD45+ cells was observed as a trend over all experiments (V=11.6 ± 1.7 SEM, Ca/N=17.9 ± 2.8; p=0.065). Notably, in HIS-BRGS mice treated with Ca alone, there was a significant decrease in overall spleen size that was reflected as both a decrease in the number of mouse and human CD45+ cells (**Supplemental Figures 2B, C**). We also observed a significant reduction in the number of mCD45+ cells (V=44.8 ± 8.3e6 vs Ca=12.0 ± 1.8e6; p=0.0012), but not in the hCD45+ cells, in the combination treated mice, suggesting the anti-human PD-1 Ab was rescuing the hCD45+ T cells. Indeed, the N treated group had the highest number of hCD45 cells in the spleens (**Supplemental Figure 2C**). This decrease was not seen in either the LNs or the TILs, suggesting it is not due to a systemic toxic effect (**Supplemental Figures 2B, C**).

Among the human leukocytes, we observed increased T cells (hCD45^+^CD3^+^) in both the spleen (p<0.0001) and TILS (V=52.9 ± 5.2, Ca/N=67.4 ± 5.0; p=0.048) in the combination-treated mice compared to untreated controls (**Figure 2B**), with a majority (~70-80%) of CD4^+^ T cells in both tissues, as is common in human blood and tissues (**Figure 2C**). There is a trend of increased CD8 T cell numbers in TILs of combination-treated mice relative to C alone (p=0.063) or V (p=0.053). Alternatively, C alone had the lowest T cell numbers but highest CD4 frequencies (82.7 ± 2.6%) in the TILs of any cohort (**Figures 2B,C**). Although B cells (CD19^+^ or CD20^+^) were prevalent in both lymph organs, they were highly underrepresented in the TILS, a finding that we and others have observed in this system previously (27, 29, 37) (**Figure 2D**). We also measured the frequency of myeloid lineage cells (CD3^-^, CD19^-^, CD11b^+^, CD11c^+^, CD14^+^ or CD33^+^) in both the spleen and in the TILS (**Supplemental Figure 2A**) and found no differences among any treatment groups, although a higher proportion of myeloid cells was observed in TILs relative to spleens (**Figure 2E**). The frequency of major histocompatibility (MHC) Class II (HLA-DR^lo^) cells within the myeloid population was similar among all treatment groups.

### Increased activated and cytotoxic CD4+ T cells in TILs treated with Ca/N

**Figure 3:**
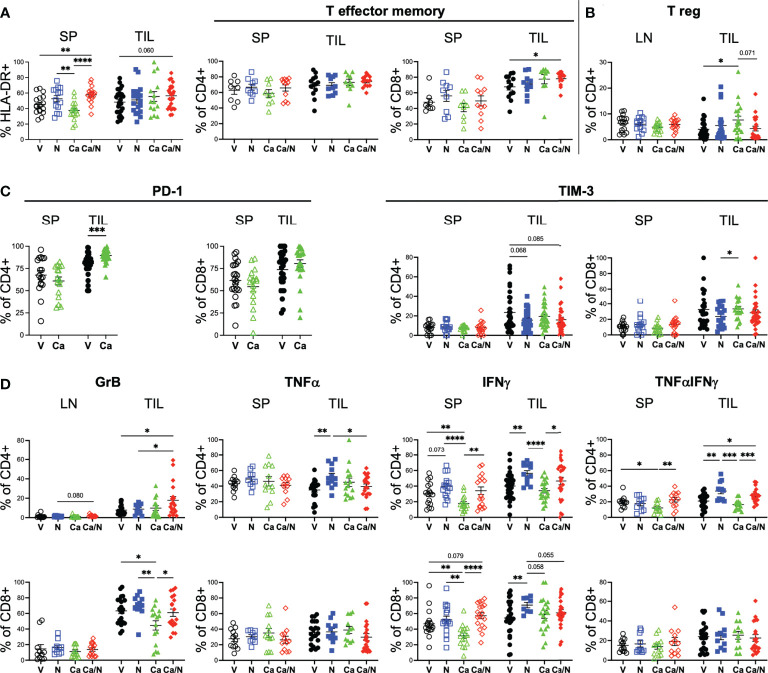
Increased activated CD4+GrB+TNFα+IFNγ cytotoxic T cells in CRC TILs of mice treated with cabozantinib and nivolumab. Frequencies of activated HLA-DR+ **(A**, left panel**)** T cells, CCR7-CD45RO+ CD4+ and CD8+ T effector cells **(A**, right panel**)**, T regulatory cells **(B)**, CD4+ and CD8+ T cells expressing inhibitory receptors PD-1 and TIM-3 **(C)**, and CD4+ (upper) and CD8+ (lower) T cells **(D)** with intracellular expression of cytotoxic Granzyme B (left panels), TNFα or IFNγ alone (middle panels) or co-expressing TNFαIFNγ (right panels) in spleens (SP), lymph nodes (LN) or TILS of HIS-CRC-BRGS mice. All data were determined using flow cytometry. Expression of TNFα and IFNγ were determined following overnight stimulation with cell stimulation in the presence of Golgi Block for the last four hours. V=untreated, N=nivolumab alone, Ca=cabozantinib alone and Ca/N=combination cabozantinib and nivolumab. P-values indicate ANOVA multiple comparisons with Welch’s correction: *p < 0.05, **p < 0.01, ***p < 0.001, ****p < 0.0001 or as indicated by number.

In both the spleen and the TILs, the T cells in the combination-treated HIS-BRGS mice had increased HLA-DR expression relative to the V cohort, a phenotype consistent with T cell activation (**Figure 3A** and **Supplemental Figure 3A**). We further assessed the activation status of the human T cells by measuring the frequency of effector memory (CD45RO^+^, CCR7^-^) T cells, a T cell subset associated with active immune responses that are generated following expansion from either naïve or memory T cells. Consistent with more activated T cells in the combination-treated groups we found increased frequencies of the effector memory CD8^+^ T cells in the TILs but not the spleens (**Figure 3A** and **Supplemental Figure 3B**). As an assessment of immunomodulatory T cell function, we quantified the frequency of regulatory T cells (Tregs, CD4^+^CD25^+^FoxP3^+^) in the LNs, where we observe clear Treg populations in our HIS-BRGS mice (**Figure 3B** and **Supplemental Figure 3E**), and TILs. Although we saw little difference among Tregs in the LNs, we did observe an increase in the frequency of Tregs among CD4^+^ T cells in the TILs of mice treated with Ca alone, but this increase was abrogated with N co-administration. As a further indication of T cell activation and exhaustion status, we also measured the frequency of both PD-1 and TIM-3 inhibitory receptors on the CD4 and CD8 T cells in the spleen and TILs (**Figure 3C**). We have previously reported that N blocks the binding of several anti-PD-1 detecting Ab clones (27, 37). Therefore, we restricted this analysis to the V and Ca single-agent groups. Interestingly we found that treatment with Ca alone resulted in a higher frequency of CD4^+^ PD-1^+^ T cells in the tumor (V=80.3 ± 2.2, Ca =89.7 ± 1.3; p=0.006), but not the spleen, and that there was a higher frequency of PD-1^+^ T cells in the TILs relative to the spleen, regardless of treatment. Alternatively, there was a trend for decreased expression of TIM-3 on the CD4+ T cells in the combination-treated TILs, indicative of less “exhausted” PD-1^+^, TIM-3^-^ T cell pool. Interestingly, differences in PD-1 and TIM-3 inhibitory receptor expression in the combination-treated TILs was restricted to the CD4+ T cells and not observed in the CD8+ T cells.

We also assessed whether the increased activated T cell populations were associated with increased cytotoxic T cell function. Using intracellular cytokine staining, we found significantly increased production of Granzyme B (GrB), and TNFα^+^IFNγ^+^ double-producing CD4^+^ T cells in the TILS of the HIS-BRGS mice treated with the combination (**Figure 3D**, GrB: V=7.7 ± 1.1, Ca/N=17.9 ± 3.9; IFNγ+TNFα+: V=20.9 ± 2.0, Ca/N=28.2 ± 2.2, p=0.020). The increase in both TNFα and IFNγ were observed in both the single agent N, and combination treated cohorts suggesting the effect was driven by the ICB therapy. However, the increase in GrB production among CD4^+^ T cells required Ca along with N. Interestingly, this effect was limited to the CD4^+^ T cells, as the presence of Ca alone actually decreased the frequency of CD8^+^ T cells expressing GrB. ICB is often reported to increase the frequency of CD8^+^IFNγ^+^ T cells. In our study, we observed this increase with single agent N, however, the co-administration of Ca appeared to offset this production. Furthermore, the activation of the IFNγ pathway was significantly reduced among CD4^+^ T cells in the mice treated with Ca alone. Thus, the combination treated mice demonstrate increased human T cell infiltration, with several signatures of increased activated and cytotoxic T cells, although Ca alone appears to have unique immune-modulating effects on T cells including upregulation of PD-1 and increased Tregs.

### Cabozantinib increases major histocompatibility complex molecules and PD-L1 expression on human CRC PDX tumors in the presence of a HIS

**Figure 4:**
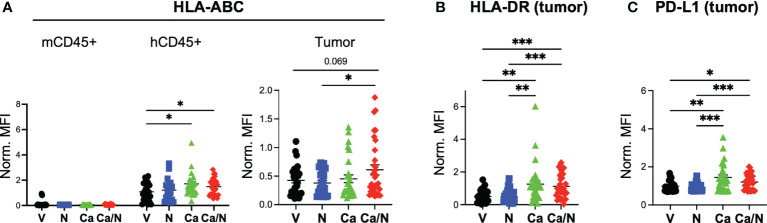
Increased MHC Class I, Class II and PD-L1 expression on CRC MSS tumors explanted from HIS-BRGS mice treated with cabozantinib, alone, and in combination with nivolumab. Mean fluorescence intensity (MFI) of MHC Class I (HLA-ABC, **A**), MHC Class II (HLA-DR, **B**) and PD-L1 **(C)** on mCD45-,hCD45-, EpCam+ CRC MSS tumor cells explanted from HIS-BRGS mice treated with V=untreated, N=nivolumab alone, Ca=cabozantinib alone and Ca/N=combination cabozantinib and nivolumab. Expression of HLA-ABC MHC class I is shown on both mCD45+ cells and hCD45+ cells, as negative and positive controls, respectively **(A)**. P-values indicate ANOVA multiple comparisons with Welch’s correction: *p < 0.05, **p < 0.01, ***p < 0.001, or as indicated by number.

As a multi-target TKI, Ca has pleiotropic effects on the immune system and tumors themselves. Studies in CRC mouse models have demonstrated changes in expression of antigen presentation molecules on tumors themselves (38), likely due to suppression of *AXL* that suppresses MHC Class I expression. We examined whether the drug altered expression of human HLA Class I (HLA-ABC) and class II (HLA-DR) on the tumor cells themselves in our HIS-mouse model. Using flow cytometry, we identified tumors as mCD45^-^, hCD45^-^, EpCAM^+^ in the tumor cell suspensions and quantified the mean fluorescence intensity (MFI) of HLAs on the tumors as well as the mCD45^+^ and hCD45^+^ cells in the TILs, as negative and positive controls, respectively (28). We observed increased expression of HLA-ABC on both the hCD45^+^ and the tumor cells with combination treatment, and the Ca alone appears to play a significant role in this upregulation (**Figure 4A**). Similarly, MHC Class II HLA-DR was upregulated as a result of Ca alone, or with N, in tumor cells (**Figure 4B**). Consistent with an IFNγ-driven upregulation of MHC molecules (39, 40), we also observed increased expression of PD-L1 on tumor cells with Ca alone and in combination with N (**Figure 4C**). Thus, Ca modulates both the expression of antigen presentation and inhibitory receptor molecules on the CRC tumors.

### Increased IFNγ and TNFα immune response to CRC tumor in HIS-BRGS mice with Ca/N compared to cobimetinib and atezolizumab (Co/A) treatment

Co has failed in phase III CRC clinical trials when tested in combination with A, an anti-PD-L1 monoclonal Ab. As a predictor for clinical success, we decided to directly compare tumor immune responses of Co in combination with A (Co/A) to the combination of Ca/N, utilizing the preclinical HIS-BRGS model. To reduce experimental variability, we chose to implant the same MSS-CRC PDX (CRC307P) into HIS-BRGS mice that had been generated with the same donor CB (**Supplemental Tables I** and **II**). We included single agent A and Co cohorts, as we had no previous data for these single agent treatments (**Supplemental Figure 4A**). Tumor growth measurements, including tumor volume, SGR, and tumor weights, again reflected the ability of these TKIs to slow the growth of PDXs in immunodeficient mouse models, as both combinations and Co alone greatly inhibited tumor growth (**Supplemental Figure 4B**).

**Figure 5:**
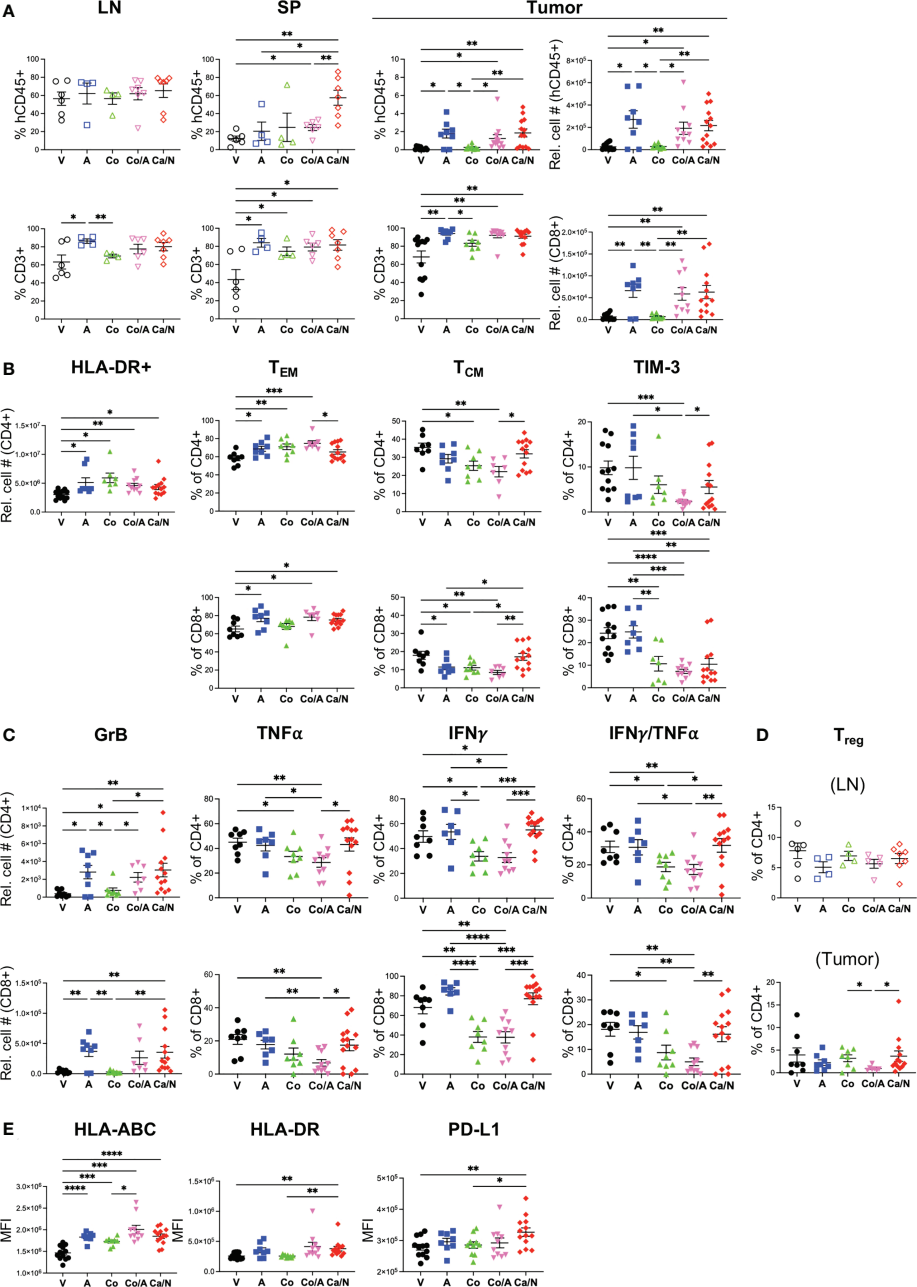
Increased central memory and cytotoxic T cell frequencies in CRC PDX MSS tumors explanted from HIS-BRGS mice treated with cabozantinib/nivolumab (Ca/N) combination relative to those treated with cobimetinib/atezolizumab (Co/A). Phenotypes of human immune system in LN, spleen (SP) and TILs (CU-CRC307P) from HIS-BRGS mice treated with nothing (V), atezolizumab **(A)**, cobimetinib (Co), combination Co/A or Ca/N: frequencies and/or numbers of **(A)** hCD45+ (Top row) and human T cells (CD3+, bottom row), **(B)** T cell populations (CD4+ top, CD8+ bottom) in the CRC307P TILS: activated HLA-DR+ (left panel), effector memory (CCR7-, CD45RO+, left middle panel), central memory (CCR7+, CD45RO+, right middle panel) and exhausted TIM-3+ (right panel); **(C)** Cytotoxic T cells (CD4+ top, CD8+ bottom) expressing Granzyme B (left panels) ex vivo or TNFα (left middle panels), IFNγ (right middle panels) or both TNFαIFNγ (right panels) following overnight cell stim and 4 hour Golgi block; **(D)** T regs (FoxP3+, CD25+) in LN (upper) and tumor (bottom); and **(E)** expression (MFI) of MHC class I (HLA-ABC, left), class II (HLA-DR, middle) and PD-L1 (right) on CRC307P EpCAM+ tumor cells. P-values indicate ANOVA multiple comparisons with Welch’s correction: *p < 0.05, **p < 0.01, ***p < 0.001, ****p < 0.0001 or as indicated by number.

As TKIs have been previously shown to affect tumor growth of CRC PDXs in mice but are not effective as single agents in the clinic, we chose to focus our analysis on the immune response to the PDX in the combination treatment cohorts. First, we compared the human immune responses of the combination Ca/N cohort to the V treated cohort. Consistent with the previous CRC combination Ca/N experiments, we observed increased human hematopoietic (hCD45^+^) and human T cells in the spleens and TILs of mice treated with Ca/N relative to V control (**Figure 5A**). In the TILs, this increase was most pronounced as increased CD8^+^ T cells per gram of tumor. Similarly, we observed an increase in activated (HLA-DR^+^) T cells and more effector memory CD8^+^ T cells in the TILs in the combination Ca/N group, relative to V control (**Figure 5B**). In this experiment, the frequency of TIM-3 expression was unchanged in CD4^+^ T cells, but greatly reduced in the CD8^+^ T cells exposed to combination Ca/N. Further, as was seen previously, there were more GrB producing CD4^+^, and in this experiment also CD8+ T cells in the TILs of the combination Ca/N treated mice (**Figure 5C**). We did not observe an increase in the TNFα^+^, IFNγ^+^ or TNFα^+^IFNγ^+^ double producing cytotoxic T cells, of either the CD4+ subset, as observed previously for TNFα+ or double-producers, or the CD8+ T cells in the combination Ca/N treated TILs relative to the V control. In this experiment both the V and single agent A treated mice had significantly large fractions of cytotoxic cytokine producing T cells, similar to the levels of the combination Ca/N treated mice. The differences observed between combination Ca/N treated tumors and V followed the same trend as previous experiments, with similar production of cytotoxic cytokines among CD8^+^ T cells and slightly higher among CD4^+^ T cells. As before, we also did not observe a difference in the frequency of CD4^+^ Tregs in the LNs or TILs of combination Ca/N treated mice (**Figure 5D**). Similarly, the combination Ca/N treatment induced upregulation of both MHC Class I and II (HLA-ABC, HLA-DR), as well as PD-L1 expression on tumor cells treated with combination Ca/N relative to the controls (**Figure 5E**).

Having defined a consistent immune response from the combination Ca/N treatments, we wanted to compare this response to the combination of Co/A cohort to determine if we could expect distinct clinical outcomes. In our CRC307P-bearing HIS-BRGS model, we observed increased splenic and tumor human hCD45^+^ cells more so in the combination Ca/N than the Co/A cohort (**Figure 5A**). However, the increase in human T cells was detected in both combination cohorts as well as the A single agent group, suggesting the ICB was responsible for this increase (**Figure 5A** and **Supplemental Figure 4C**). We next wanted to address whether T cell responses were similar, or not, between the combination Ca/N and Co/A cohorts. Although upregulation of HLA-DR, an “early” activation marker, were similar among T cells in the tumors of mice receiving either combination treatment, we identified other T cell activation signatures that were quite distinct among these groups (**Figure 5B**). Specifically, the frequency of central memory CD4^+^, and even more so, CD8^+^ T cells was significantly decreased in mice treated with Co or A, and with a more pronounced effect with the combination. We also observed fewer TIM-3+ CD4^+^ and CD8^+^ T cells in the Co (for CD8^+^ only) or combination Co/A treated TILs, especially compared to the V or A single-agent groups. As in the combination Ca/N treated mice, we observed increases in GrB producing T cells in tumors of mice treated with A, either as single-agent or in combination with Co (**Figure 5C**). Alternatively, the Co treatment, alone or with A, resulted in much lower frequency of CD4^+^ or CD8^+^ T cells with TNFα, and even more so IFNγ production in the tumor (CD4+ IFNγTNFα+: Co/A=17.2 ± 3.1, Ca/N=31.8 ± 4.1, p=0.0097). Interestingly, combination Co/A had fewer Tregs in the TILs than the combination Ca/N group (**Figure 5D**, Co/A=0.95 ± 0.20, Ca/N=3.7 ± 1.2, p=0.036).

Both combination treatments had more human T cells in the TILs, but more of these T cells in tumors of mice treated with Ca/N were of the central memory phenotype, with higher production of TNFα and IFNγ cytotoxic cytokines in the combination Ca/N cohort, yet fewer Tregs in the combination Co/A cohort. Additional analyses showed similar upregulation of antigen presentation molecules HLA-ABC and HLA-DR on tumors from both combination groups (**Figure 5E**). However, the increase of PD-L1 was observed only in the combination Ca/N group. Interestingly, both targeted drugs appeared to upregulate the expression of PD-1 on the tumor itself (**Supplemental Figure 4D**). In our assessment of myeloid lineage, we observed an increase in plasmacytoid dendritic cells (pDCs: Lin- (CD3,19,56,11b,14,11c,33) hCD45^+^ HLA-DR^hi^, in the combination Ca/N cohort specifically that correlates with ICB treatment, reduced HLA-DR+ human myeloid cells in the Co/A treated-tumors, correlative with more myeloid-derived suppressor like cells, and decreased mCD45^+^ cells, which are all myeloid lineage in this model, with Co alone (**Supplemental Figure 4E, F**).

### CD4+ T cell immunotypes correlate with reduced tumor growth in HIS-BRGS models

**Figure 6:**
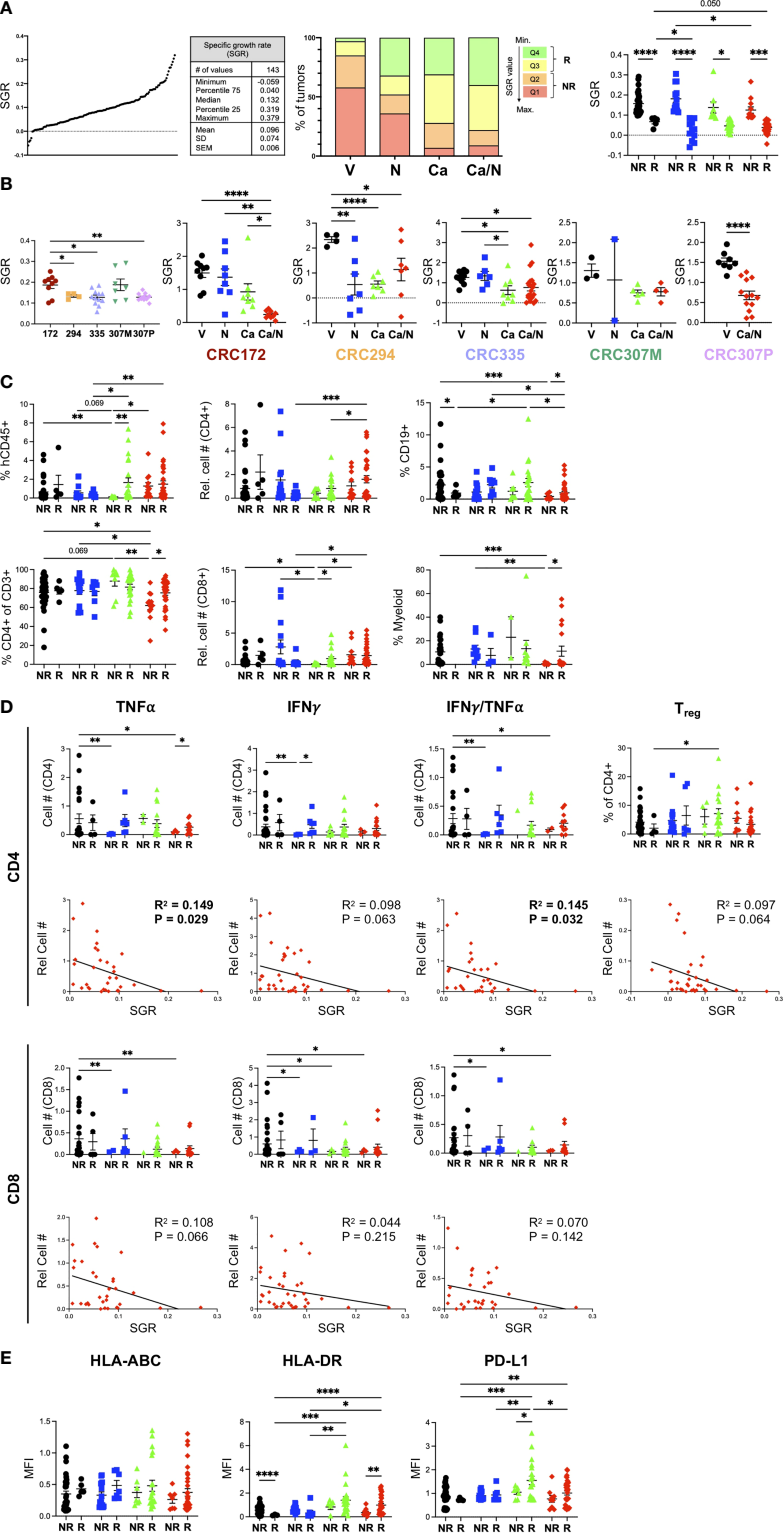
Increased CD4+ TNFα+IFNγ+ signature correlates with smaller CRC MSS tumors from HIS-BRGS mice treated with Ca/N combination. **(A)** SGR were determined for each of 5 CRC PDX MSS tumors studied in HIS-BRGS mice: distribution of SGRs among all PDX tumors (left panel), frequencies of SGR according to quartiles (middle panel) and to responder (Q3+Q4) or non-responder (Q1+Q2) assignments (right panel) among four treatment cohorts. **(B)** Tumor growth, as measured by SGR, in untreated (left panel) and treatment responses (right panels) among 5 CRC MSS PDX studied in HIS-BRGS mice. **(C)** Human immune populations among non-responder and responder tumors from different treatment groups. **(D)** Number of TNFα+, IFNγ+ or IFNγ+TNFα+ cytotoxic CD4+ (upper panels) and CD8+(lower) panels in non-responder and responder (upper) and in correlation with the SGR (lower) of CRC MSS PDXs from HIS-BRGS mice in four treatment groups. SGR correlations are from combination treatment groups (red) only. **(E)** Expression, as indicated by MFI, of MHC Class I (HLA-ABC, left), MHC Class II (middle) and PD-L1 (right) on EpCAM+ MSS-CRC PDX explanted from HIS-BRGS mice by responder/non-responder tumor status and according to treatment groups. For all panels, colors indicate different treatment groups: (black- vehicle, blue-nivolumab, green-cabozantinib, red- combination). For column graphs, P-values indicate ANOVA multiple comparisons with Welch’s correction: *p < 0.05, **p < 0.01, ***p < 0.001, ****p < 0.0001. For SGR correlations, linear regression analysis was used to assess the correlation (R2) and significance, as indicated by p-value. Values in bold indicated statistical significance with a p < 0.05 cut-off.

Having established an “immune signature” that correlates with combination Ca/N treatment, we wanted to determine if these “immunotypes” correlated with tumor growth inhibition in our HIS-BRGS model. Tumor growth in all models (mouse, patients, HIS-mice) has inherent variability that can be dependent upon tumor clones and microenvironment, including an immune reaction. The SGR takes into account individual differences in the starting size of tumors and is independent of the final tumor measurement date. Using the SGR to quantitate the growth of each tumor in our studies revealed a range and normal distribution of tumor growth rates across all PDXs and treatment groups (**Figure 6A**). From these data, we allocated the tumors into growth “quartiles” with the fastest tumors (i.e., highest SGR) in quartile 1 (Q1) and the slowest growers in quartile 4 (Q4). We found the highest frequency of slow growing tumors (Q4 and Q3) in the Ca alone group and in the combination Ca/N treatment group.

For each PDX, we normalized the SGR across treatment groups to determine the response to treatment for that PDX (**Figure 6B**). As expected, the PDXs had variable responses to treatments: CRC294 responded to single agent N or Ca, CRC335 had minor, while CRC172 and CRC307P had the strongest, responses to combination Ca/N therapy. Although the metastatic lesion of the 307 PDX (CRC307M) had a marginal response to Ca/N therapy, the PDX obtained from the primary lesion (CRC307P) had a very strong response. These data support the expected outcome of differential responses to treatment depending upon both individual tumor characteristics, immune responses, and tumor progression (primary tumor vs metastasis).

We used the SGR as a measurement to define individual tumors as “responding, Q4+Q3)” or “non-responding (Q2+Q1)” and to evaluate the correlation between individual immune parameters and the tumor growth (**Figure 6A**). Using this approach and looking at differences among tumors in the combination Ca/N treated mice, we found a trend of increased human CD45^+^ in the responding tumors compared to the faster-growing, non-responding tumors (**Figure 6C**). This difference was significant with a higher frequency of human CD4^+^ T cells, B cells and myeloid cells observed in the Ca/N responding tumors compared to the non-responding tumors. An increase in mouse hematopoietic cells, of the myeloid lineage in this model, correlated with responders in the Ca-treated animals (**Supplemental Figure 5A**).

We next examined the correlation of cytotoxic cytokine (IFNγ, TNFα)-secreting CD4^+^ and CD8^+^ T cells, and Tregs within responding and non-responding tumors (**Figure 6D** and **Supplemental Figure 5**). Consistent with the increases in T cell populations among all combination Ca/N treated tumors observed in **Figure 3**, we found increased TNFα+ and IFNγ^+^ CD4^+^ T cell numbers in responding tumors compared to non-responding. A linear regression analysis of the SGR with human, and more specifically T cell populations, confirmed the strongest correlation of CD4^+^ T cells with reduced tumor growth. Importantly, the number of TNFα^+^ and TNFαIFNγ^+^ CD4^+^ T cells significantly correlated with lower tumor growth rates, while the CD8 T cells showed a less drastic effect. The inverse correlation of Tregs with tumor growth rate was surprising although ICB treatment has been shown to induce proliferation of PD-1^+^ Tregs (41). We observed no differences with the frequencies or numbers of CD4+ or CD8+ effector memory or GrB^+^ CD4^+^ T cells (**Supplemental Figure 5B**). Indeed, the frequency of GrB^+^ CD8^+^ T cells was lower in the responding tumors (**Supplemental Figure 5B**), consistent with our observed reduction in this population among combination treated tumors (**Figure 3**). Increased TIM-3^+^CD4^+^ T cells correlated with larger tumors, suggesting more exhausted T cell pool is non-favorable to tumor growth inhibition (**Supplemental Figure 5B**).

We also tested the expression of HLA-ABC and HLA-DR on the tumor cells themselves among responding and non-responding tumors (**Figure 6E**). Although we observed increases of both of these molecules with Ca alone or in combination with N treatments (**Figure 4**), we found a surprising lack of correlation with the MHC Class I expression but a statistically significant benefit of increased MHC Class II expression in responding tumors (**Figure 6E**) and an association of increased MHC Class II in smaller (low SGR) tumors (28). This finding is consistent with a stronger correlation of CD4^+^ T cells in the tumor immune response. Further, we also found that higher PD-L1 expression, which we observed in the combination treated tumors, showed a trend for higher expression among responding combination-treated tumors but was only significantly increased in responding tumors for Ca single treatment.

Interestingly, this tumor growth analysis identified a strong correlation of hCD45+ cells with the responders among the single agent Ca treated tumors (**Figure 6C**). Across all PDXs we observed a significant decrease in tumor growth in this single-agent group, that we surmised was due to direct effects of drug on the tumor that is observed more in mouse models than in human patients. However, these analyses support an additional role of Ca in altering the human immune response, and in these experiments the strongest correlation was with the CD8^+^ T cells. An increase in GrB^+^ and IFNγ^+^TNFα^+^ CD4^+^ T cells with fewer central memory T cells and surprisingly fewer IFNγTNFα^+^ CD8^+^ T cells in tumor, with a decreased presence of myeloid, notably HLA-DR^lo^ cells, correlated with smaller tumors (**Supplemental Figure 6**). We conclude that Ca alone influences the human immune system in this model, with immunotypes distinct from the combination treatment group (i.e., TNFαIFNγ^+^ CD4^+^ T cells).

## Discussion

Utilizing an HIS-BRGS mouse model, we provide pre-clinical evidence in support of the Ca/N combination for MSS-CRC. Over 5 independent CRC MSS PDX models, we found that Ca alone and in combination with N resulted in reduced tumor growth. In addition to the tumor growth data, we gathered abundant flow cytometric data interrogating the HIS from both immune organs and the human tumor. Using this approach, we showed that the Ca/N combination resulted in changes in the immune response, noted by consistent increases in human T cells (hCD45^+^CD3^+^) in the spleens and TILs, with increases in both CD4^+^ and CD8^+^ T cells. To determine changes in T cell activation state and function we measured the following molecules on the T cells within both the peripheral lymph organs and the TILs: activated T cells (HLA-DR), effector/memory T cells (CCR7/CD45RA or CD45RO), inhibitory receptors that are initially upregulated upon activation and then increase with exhaustion (PD-1, TIM-3), and secretion of cytotoxic GrB, TNFα and IFNγ molecules as measurements of function. In our studies in mice treated with combination Ca/N, the immune population was characterized by activated T cells in the tumors, with increased HLA-DR expression, increased frequencies of effector memory CD8^+^ T cells, and most notably a population of cytotoxic CD4^+^ T cells showing increased production of GrB, TNFα and IFNγ. Additionally, there was a trend for decreased expression of TIM-3 on the CD4^+^ T cells in the combination treated mice, which was indicative of a less “exhausted” T cell state with this regimen. Moreover, we observed enhanced antigen presentation with upregulation of both MHC class I and II (HLA-ABC, HLA-DR) molecules and increased expression of PD-L1 on tumor cells in the combination treated mice.

By evaluating multiple immune parameters, we were able to identify distinct immunotypes that correlated with disease response by treatment regimen. We found that treatment with ICB alone or in combination significantly increased production of TNFα^+^IFNγ^+^ double producing CD4^+^ T cells among the TILS. However, combination treatment with Ca/N resulted in increased GrB production among CD4^+^ T cells, which is likely due to the addition of Ca. Ca is a multi-target TKI that has been previously shown to have immunomodulatory properties. Prior studies in murine colon carcinoma (MC38) cells showed that treatment with Ca led to reduced tumor proliferation and upregulation of MHC class I molecules, both on a population level and a per-cell basis, and similar to our human tumor studies (38). This study did not evaluate MHC class II, that correlates with reduced tumor growth in our study more than class I. Similarly, Ca alone resulted in increased frequency of CD8^+^ IFNγ^+^ T cells, decreased myeloid cells, and increased Tregs in both human and mouse models (38). Our work also showed increased frequencies of PD-1^+^ T cells in the tumors with Ca treatment alone. More TIM-3^-^PD-1^+^ CD4^+^ T cells in the tumor support the notion of an increased population of exhausted T cells capable of reinvigoration (42). Data from a phase II clinical trial which enrolled patients with platinum-refractory metastatic urothelial carcinoma that were treated with single agent Ca was recently published (43). Translational immune subset biomarkers were reported from this trial, with noted increases in the ratio of effector CD8^+^ T cells to Tregs as a result of treatment with Ca. Further, Ca significantly increased the expression of PD-1 and downregulated the expression of TIM-3 on Tregs, data consistent with our Ca/N cohort (43). However, in contrast to our findings and data from mouse models, Ca decreased the percentage of Tregs in the CD4^+^ T cell population in the phase II clinical trial. Similar findings were also seen in a preclinical castrate-resistant prostate cancer mouse model treated with combination Ca and ICB (44). In our HIS-mouse model we found a CD4^+^ T cell bias with Ca alone that was reversed with the CD8-favored ICB treatment. Cell depletion studies are needed to fully evaluate the impact of these agents on the tumor microenvironment. These studies are feasible with our HIS-mouse model (29).

We further correlated our immunotypes with tumor growth characteristics to evaluate which immunotypes are most associated with anti-tumor immune responses. First, we established tumors into responders and non-responders based on SGR. Using this approach, we observed distinct differences in immunotypes between responding and non-responding tumors. Tumors that were “responding” to treatment were characterized by increased frequencies of CD45+ cells and increased frequencies of TNFα^+^ and IFNγ^+^ CD4^+^ T cells. Using a linear correlation approach between immunotype and the SGR for each tumor, we observed tumor growth inhibition was most strongly correlated with higher levels of HLA-DR^+^TIM-3-TNFα^+^ and TNFα^+^IFNγ^+^ CD4^+^ T cells. Notably, the frequency of GrB^+^CD4^+^ T cells did not correlate with anti-tumor response and in fact was negatively correlated among the CD8+ T cells. Although both MHC class I and II were upregulated on tumor cells in mice treated with Ca/N combination, only HLA-DR expression correlated with smaller tumors (28). This finding is consistent with the CD4^+^ immunotype found to be associated with anti-tumor responses. The increased PD-L1 expression, along with HLA class I and II, on tumor cells treated with Ca/N is a common theme in tumor immunotherapy and is consistent with an IFNγ driven response.

To evaluate clinical relevance of our studies in the HIS-BRGS model, we compared distinct TKI/ICB combinations (Ca/N vs Co/A). This latter combination has already failed Phase III clinical trials following initial promising results (23). We compared these two regimens in the HIS-BRGS model, with the same CRC MSS PDX and in HIS-mice prepared from the same CB to reduce experimental variability. Although tumor growth was suppressed by both combinations and single agent TKI, we were able to detect both similarities, and more importantly, differences in immunotypes in tumors from mice treated with the different TKI/ICB regimens. Both combination treatments resulted in increased human T cells in the tumors with an activated HLA-DR^+^ phenotype. However, there were significantly higher frequencies of CD4^+^ and CD8^+^ central memory T cells among mice treated with Ca/N and more of these T cells were positive for TNFα and IFNγ Type 1 cytokines. These data imply a defect in Tcf-1 transcription factor function upon exposure to Co, and thus may correlate with a less sustained T cell response, as central memory T cells are able to continually produce more effectors (45). Similarly there was higher upregulation of the MHC class I and II molecules, known to be associated with the increased IFNγ signature with Ca/N (46). Even more so, there was an increase in PD-L1 expression observed in the Ca/N group, but not Co/A, which may be related to the different target of N (PD-1) and A (PD-L1). For this PDX, both treatment groups resulted in upregulation of PD-1 on the tumor itself. These differences provide potential rationale for improved efficacy of Ca/N in clinical trials. There is considerable interest in exploring different combination TKI/ICB regimens to treat refractory MSS-CRC, as new drug development in this subset of patients is urgently needed. There are multiple ongoing clinical trials in various stages of progress and utilizing a variety of agents in this setting.

The HIS-BRGS mouse model has a unique set of limitations which need to be considered in the interpretation of the data generated in this research. First, HIS-mouse models are known to have significantly reduced percentages of human myeloid cells compared to both T- and B-lymphocyte populations and when compared directly to that of human blood (47–49). We have observed higher frequencies of myeloid cells in the tissues and in tumor relative to the lymph nodes or the spleen (27, 37). In an effort to overcome the limitation of an underrepresented myeloid population, several groups have developed transgenic mouse models expressing human cytokines which support myelopoiesis. The NOD-*Scid Il2rγnull-3/GM/SF (NSGS*) *(* 50) model is a transgenic mouse model which has been developed and one in which human tumors have been shown to grow successfully, which has lent to its utility in preclinical immunotherapy studies (51). Another limitation of this model is the HLA-mismatch between the PDX-derived tumor and CB-derived immune system (27). There are efforts underway to develop a completely matched thymus-HIS-tumor humanized mouse avatar, however, such a model is at the moment technically limited. It is important to consider the need to test tumor growth in improved HIS-mouse models that may contain reduced innate immunosuppression.

In conclusion, we were able to demonstrate tumor growth inhibition and augmentation of the immune response with increased immunogenicity in HIS-BRGS mice bearing human MSS-CRC PDX tumors when treated with Ca/N. These compelling preclinical data have led to the development of a clinical trial for patients with metastatic/refractory CRC that are pMMR/MSS in the third line setting and beyond (NCT04963283). This is a phase II, single-arm, open-label study of cabozantinib in combination with nivolumab and is open for enrollment at the University of Colorado. Forty-six patients will be enrolled to this study so we will be able to confirm if the immune response/modulation observed with this treatment combination translates into clinical benefit and improved outcomes for MSS-CRC patients.

## Data availability statement

The raw data supporting the conclusions of this article will be made available by the authors, without undue reservation.

## Ethics statement

The animal studies were reviewed and approved by University of Colorado Anschutz Institutional Animal Care and Use Committee (00021). All acquisition of human tumor samples were consented under IRB approvals (08-0439) as indicated in Materials and Methods.

## Author contributions

JL, AL, and JM-J wrote the first draft of the manuscript. JL, AL, JM-J, CL, WM, and TP contributed to the conceptualization and design of this study and data analysis and interpretation. JL, JM-J, SH, JS, NN, ML, SB, BY, MM, BF, KJ, RP, and TP contributed to collection and assembly of data. PB performed the statistical analysis. AL provided technical support and project administration. All authors contributed to manuscript revision, read, and approved the submitted version.

## Funding

Exelixis supplied cabozantinib and funded this study, with TP as Principal Investigator. This work was partially supported by the University of Colorado Cancer Center Support Grant (P30CA046934-25). This study was supported in part by the National Institutes of Health P30CA06934 Cancer Center Support Grant with use of the PHISM (Pre-clinical Human Immune System Mouse Models) Shared Resource, RRID: SCR_021990 and Flow Cytometry Shared Resource, RRID: SCR_022035.

## Acknowledgments

The authors would like to thank the patients that consented the use of their tumors for this study. We would also like the thank Christine Childs, and others in the University of Colorado Anschutz, Flow Cytometry Core, and the entire staff of the Office of Laboratory Animal Resources.

## Conflict of interest

Exelixis supplied cabozantinib and funded this study, with TP as Principal Investigator, but had no role in study design, data collection, data analysis, data interpretation, or writing of the report. WM is the principal investigator of a phase II clinical trial of cabozantinib in patients with refractory metastatic colorectal cancer that is sponsored by Exelixis NCT03542877. AL is the principal investigator of a phase II clinical trial of cabozantinib in combination with nivolumab in metastatic colorectal cancer which is co-sponsored by Bristol Myers Squibb and Exelixis NCT04963283.

The remaining authors declare that the research was conducted in the absence of any commercial or financial relationships that could be construed as a potential conflict of interest.

## Publisher’s note

All claims expressed in this article are solely those of the authors and do not necessarily represent those of their affiliated organizations, or those of the publisher, the editors and the reviewers. Any product that may be evaluated in this article, or claim that may be made by its manufacturer, is not guaranteed or endorsed by the publisher.
