# Supplementary material for: Cabozantinib sensitizes microsatellite stable colorectal cancer to immune checkpoint blockade by immune modulation in human immune system mouse models
Source: Front Oncol. 2022 Nov 7;12:877635. doi: 10.3389/fonc.2022.877635 (PMC9676436; doi:10.3389/fonc.2022.877635)
Supplement: Supplementary file 1 [file DataSheet_1.docx]

**Supplementary Methods**

HIS-CB-BRGS mice studies

Lightly irradiated (300 rad) newborn BALB/c-Rag2nullIl2rγnullSirpaNOD (BRGS) pups were injected with 200,000-600,000 CD34+ cells that had been in vitro differentiated in the presence of Stem Cell factor (SCF), Flt3L and IL-6 cytokines for 4-6 days. The HIS-BRGS mice were bled between 8-11 and 14-16 weeks to measure human chimerism by flow cytometry with the following antibodies (Abs): mCD45, hCD45, hCD3, hCD8, hCD19 or CD20, hPD-1. Once chimerism was confirmed, MSS CRC PDX models (CUCRC172, CUCRC294, CUCRC307, and CUCRC335 **[see Supplemental Table I]**) were implanted, in independent experiments, into both flanks of HIS-BRGS mice as described previously.(Capasso et al., 2019, Marin-Jimenez et al., 2021),(Marin-Jimenez et al., 2021) All studies were conducted with prior approval from the University of Colorado Animal Care and Use Committee and in a facility accredited by the American Association for Accreditation of Laboratory Animal Care.

Humanized Mice, tumor injections and tissue harvest

Mice were allocated into four groups based upon human chimerism as determined in the blood at 14-16 weeks post engraftment **(Supplemental Figure 1)**. The following treatments were begun when tumors reached 150-300mm3: vehicle, cabozantinib (15 mg/kg, PO, QDx5), nivolumab (15 mg/kg, IP, QW), and the combination. Mice were monitored twice weekly for toxicity and tumor volumes were measured with digital calipers using the Study Director software (Studylog Systems, South San Francisco, CA). At end of study, specific growth rates (SGRs) were calculated (SGR=ln(V2/V1)/(t2-t1) to indicate relative tumor growth rates for comparison of mice harvested in different experiments and at different timepoints. Mice were euthanized in cohorts of 4-8 mice per group according to health and tumor size.

At end of study (see Supplemental Table II) the HIS-BRGS mice were euthanized and lymph node (LN), spleen and tumor tissues harvested and prepared into single cell suspensions as described previously,35 with the following exception: digestion of the CRC307M and CRC307P PDXs were performed using a modified protocol on a Miltenyi Gentlemacs Dissociator. LN and spleen cell suspensions were enumerated using a hemocytometer. In all experiments, excised tumors were weighed, and 1/4 of the tumor removed for both immunohistochemistry (IHC) and genomic assays. The remaining tumor tissue was mechanically digested, albeit less so when using the Miltenyi Gentlemacs, in a serum-free RPMI media containing Liberase DL (50 µg/ml) and incubated at 37°C for 25’ (CRC172, CRC294 and CRC335), as described previously,(Capasso et al., 2019, Marin-Jimenez et al., 2021) or incubation for 40’ on Gentlemacs (CRC307M and CRC307P), with the program defined by tumor characteristics. In all cases, tumors were washed with RPMI media containing 10% heat inactivated FBS, DNAase, 100 U/ml penicillin, 100 μg/ml streptomycin and non-essential amino acids to stop digestion and resuspended in IMDM media containing 10% FCS with DNAase.

Flow cytometry and Analysis

Human chimerism was determined in the lymph organs and presented as a ratio of human hematopoietic cells of total hematopoietic. Mice with insufficient chimerism (defined as LNs <80% and spleens with <20% T cells) at end of study were excluded from analysis. For intracellular staining samples for FoxP3, the BioLegend Transcription Factor staining kit was used as per manufacturer’s instructions. For detection of TNFα and IFNγ intracellular cytokines, the samples were stimulated overnight with Invitrogen Cell stimulation cocktail with BD Golgiplug added the final 4 hours, as described previously.(Capasso et al., 2019, Marin-Jimenez et al., 2021) A 1% paraformaldehyde solution was used for fixation and a Saponin (1X PBS, 0.5% BSA and 0.5% Saponin) solution was used for permeabilization and intracellular staining.

Relative cell counts were collected for gated populations for each sample, multiplied by total sample volume and, for tumors, divided by weight of tumor, to compare cell numbers across samples.
