# Supplementary material for: Cabozantinib sensitizes microsatellite stable colorectal cancer to immune checkpoint blockade by immune modulation in human immune system mouse models
Source: Front Oncol. 2022 Nov 7;12:877635. doi: 10.3389/fonc.2022.877635 (PMC9676436; doi:10.3389/fonc.2022.877635)
Supplement: Supplementary file 2 [file Presentation_1.pptx]

## Slide 1
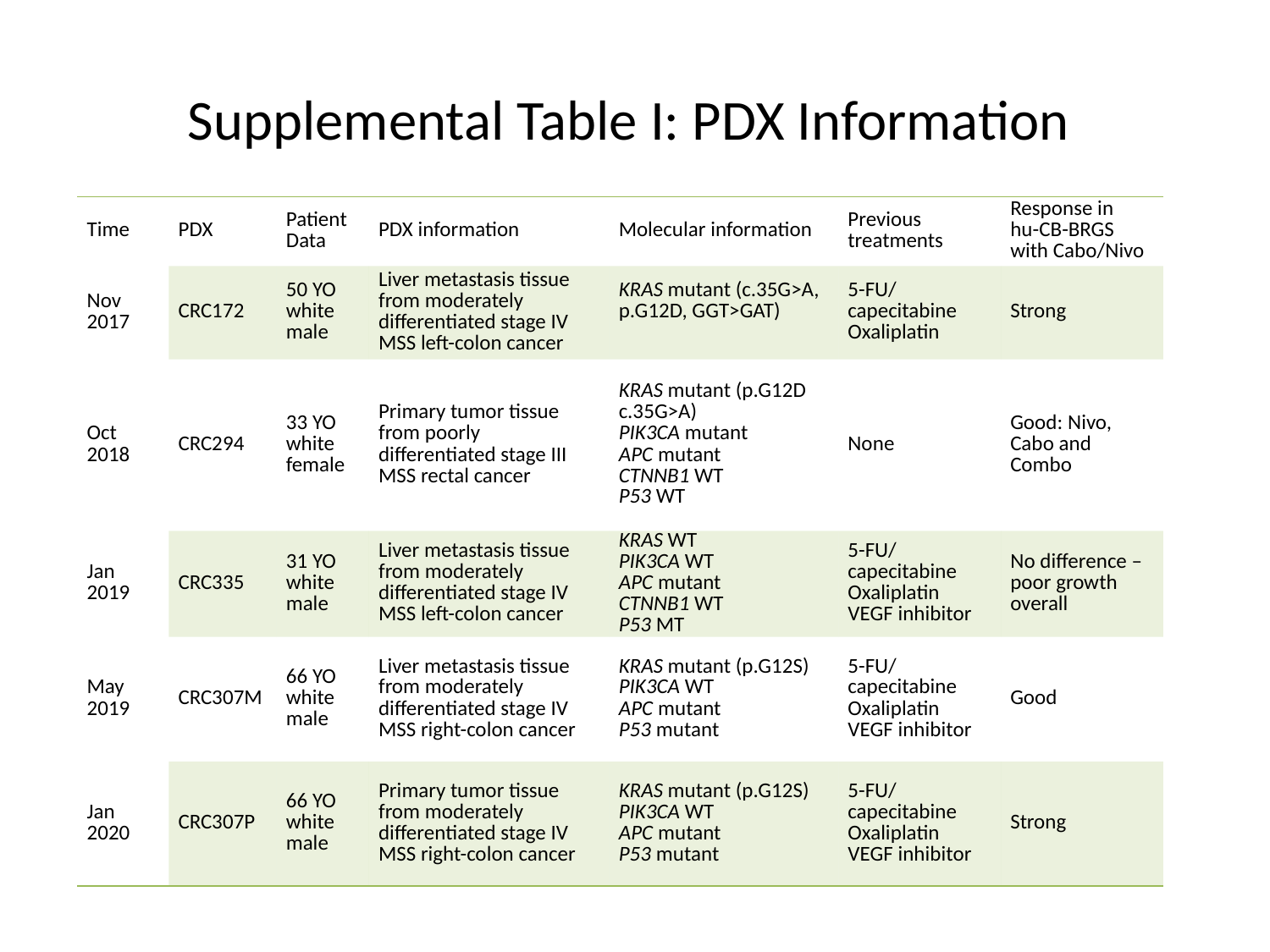

# Supplemental Table I: PDX Information
| Time | PDX | Patient Data | PDX information | Molecular information | Previous treatments | Response in hu-CB-BRGS with Cabo/Nivo |
| --- | --- | --- | --- | --- | --- | --- |
| Nov 2017 | CRC172 | 50 YO white male | Liver metastasis tissue from moderately differentiated stage IV MSS left-colon cancer | KRAS mutant (c.35G>A, p.G12D, GGT>GAT) | 5-FU/ capecitabine Oxaliplatin | Strong |
| Oct 2018 | CRC294 | 33 YO white female | Primary tumor tissue from poorly differentiated stage III MSS rectal cancer | KRAS mutant (p.G12D c.35G>A) PIK3CA mutant APC mutant CTNNB1 WT P53 WT | None | Good: Nivo, Cabo and Combo |
| Jan 2019 | CRC335 | 31 YO white male | Liver metastasis tissue from moderately differentiated stage IV MSS left-colon cancer | KRAS WT PIK3CA WT APC mutant CTNNB1 WT P53 MT | 5-FU/ capecitabine Oxaliplatin VEGF inhibitor | No difference – poor growth overall |
| May 2019 | CRC307M | 66 YO white male | Liver metastasis tissue from moderately differentiated stage IV MSS right-colon cancer | KRAS mutant (p.G12S) PIK3CA WT APC mutant P53 mutant | 5-FU/ capecitabine Oxaliplatin VEGF inhibitor | Good |
| Jan 2020 | CRC307P | 66 YO white male | Primary tumor tissue from moderately differentiated stage IV MSS right-colon cancer | KRAS mutant (p.G12S) PIK3CA WT APC mutant P53 mutant | 5-FU/ capecitabine Oxaliplatin VEGF inhibitor | Strong |

## Slide 2
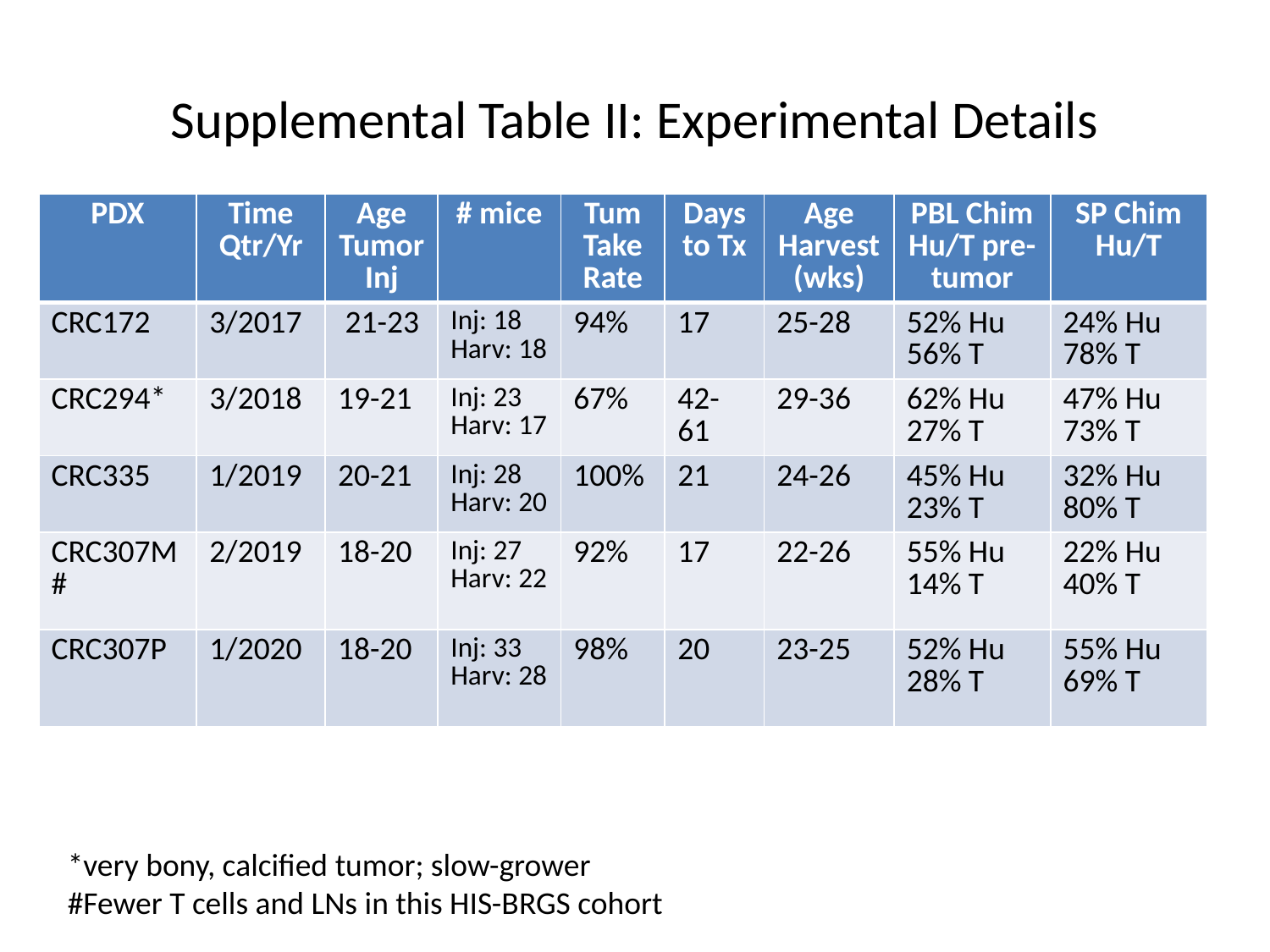

# Supplemental Table II: Experimental Details
| PDX | Time Qtr/Yr | Age Tumor Inj | # mice | Tum Take Rate | Days to Tx | Age Harvest (wks) | PBL Chim Hu/T pre-tumor | SP Chim Hu/T |
| --- | --- | --- | --- | --- | --- | --- | --- | --- |
| CRC172 | 3/2017 | 21-23 | Inj: 18 Harv: 18 | 94% | 17 | 25-28 | 52% Hu 56% T | 24% Hu 78% T |
| CRC294\* | 3/2018 | 19-21 | Inj: 23 Harv: 17 | 67% | 42-61 | 29-36 | 62% Hu 27% T | 47% Hu 73% T |
| CRC335 | 1/2019 | 20-21 | Inj: 28 Harv: 20 | 100% | 21 | 24-26 | 45% Hu 23% T | 32% Hu 80% T |
| CRC307M# | 2/2019 | 18-20 | Inj: 27 Harv: 22 | 92% | 17 | 22-26 | 55% Hu 14% T | 22% Hu 40% T |
| CRC307P | 1/2020 | 18-20 | Inj: 33 Harv: 28 | 98% | 20 | 23-25 | 52% Hu 28% T | 55% Hu 69% T |
*very bony, calcified tumor; slow-grower
#Fewer T cells and LNs in this HIS-BRGS cohort

## Slide 3
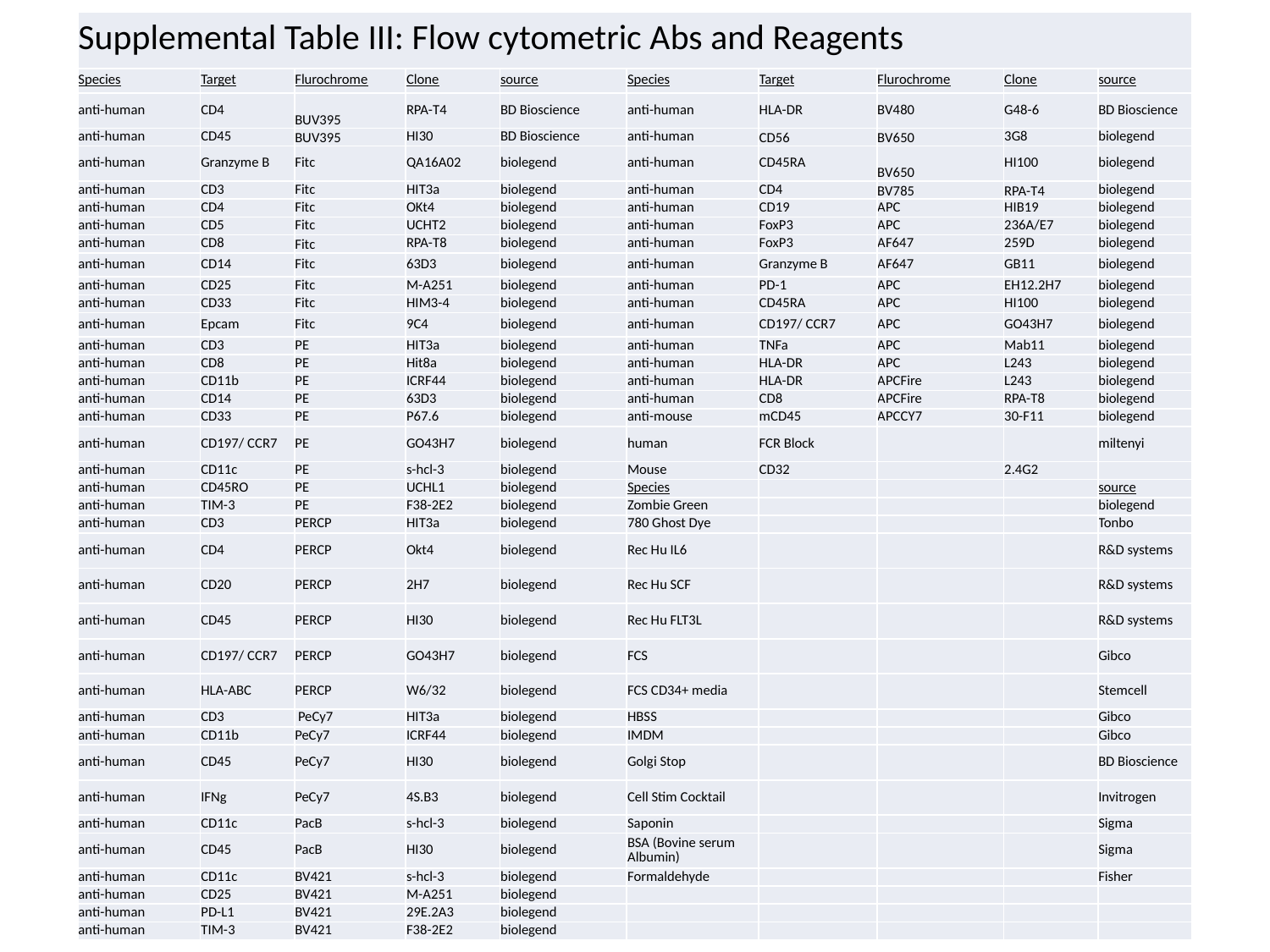

| Supplemental Table III: Flow cytometric Abs and Reagents | | | | | | | | | |
| --- | --- | --- | --- | --- | --- | --- | --- | --- | --- |
| Species | Target | Flurochrome | Clone | source | Species | Target | Flurochrome | Clone | source |
| anti-human | CD4 | BUV395 | RPA-T4 | BD Bioscience | anti-human | HLA-DR | BV480 | G48-6 | BD Bioscience |
| anti-human | CD45 | BUV395 | HI30 | BD Bioscience | anti-human | CD56 | BV650 | 3G8 | biolegend |
| anti-human | Granzyme B | Fitc | QA16A02 | biolegend | anti-human | CD45RA | BV650 | HI100 | biolegend |
| anti-human | CD3 | Fitc | HIT3a | biolegend | anti-human | CD4 | BV785 | RPA-T4 | biolegend |
| anti-human | CD4 | Fitc | OKt4 | biolegend | anti-human | CD19 | APC | HIB19 | biolegend |
| anti-human | CD5 | Fitc | UCHT2 | biolegend | anti-human | FoxP3 | APC | 236A/E7 | biolegend |
| anti-human | CD8 | Fitc | RPA-T8 | biolegend | anti-human | FoxP3 | AF647 | 259D | biolegend |
| anti-human | CD14 | Fitc | 63D3 | biolegend | anti-human | Granzyme B | AF647 | GB11 | biolegend |
| anti-human | CD25 | Fitc | M-A251 | biolegend | anti-human | PD-1 | APC | EH12.2H7 | biolegend |
| anti-human | CD33 | Fitc | HIM3-4 | biolegend | anti-human | CD45RA | APC | HI100 | biolegend |
| anti-human | Epcam | Fitc | 9C4 | biolegend | anti-human | CD197/ CCR7 | APC | GO43H7 | biolegend |
| anti-human | CD3 | PE | HIT3a | biolegend | anti-human | TNFa | APC | Mab11 | biolegend |
| anti-human | CD8 | PE | Hit8a | biolegend | anti-human | HLA-DR | APC | L243 | biolegend |
| anti-human | CD11b | PE | ICRF44 | biolegend | anti-human | HLA-DR | APCFire | L243 | biolegend |
| anti-human | CD14 | PE | 63D3 | biolegend | anti-human | CD8 | APCFire | RPA-T8 | biolegend |
| anti-human | CD33 | PE | P67.6 | biolegend | anti-mouse | mCD45 | APCCY7 | 30-F11 | biolegend |
| anti-human | CD197/ CCR7 | PE | GO43H7 | biolegend | human | FCR Block | | | miltenyi |
| anti-human | CD11c | PE | s-hcl-3 | biolegend | Mouse | CD32 | | 2.4G2 | |
| anti-human | CD45RO | PE | UCHL1 | biolegend | Species | | | | source |
| anti-human | TIM-3 | PE | F38-2E2 | biolegend | Zombie Green | | | | biolegend |
| anti-human | CD3 | PERCP | HIT3a | biolegend | 780 Ghost Dye | | | | Tonbo |
| anti-human | CD4 | PERCP | Okt4 | biolegend | Rec Hu IL6 | | | | R&D systems |
| anti-human | CD20 | PERCP | 2H7 | biolegend | Rec Hu SCF | | | | R&D systems |
| anti-human | CD45 | PERCP | HI30 | biolegend | Rec Hu FLT3L | | | | R&D systems |
| anti-human | CD197/ CCR7 | PERCP | GO43H7 | biolegend | FCS | | | | Gibco |
| anti-human | HLA-ABC | PERCP | W6/32 | biolegend | FCS CD34+ media | | | | Stemcell |
| anti-human | CD3 | PeCy7 | HIT3a | biolegend | HBSS | | | | Gibco |
| anti-human | CD11b | PeCy7 | ICRF44 | biolegend | IMDM | | | | Gibco |
| anti-human | CD45 | PeCy7 | HI30 | biolegend | Golgi Stop | | | | BD Bioscience |
| anti-human | IFNg | PeCy7 | 4S.B3 | biolegend | Cell Stim Cocktail | | | | Invitrogen |
| anti-human | CD11c | PacB | s-hcl-3 | biolegend | Saponin | | | | Sigma |
| anti-human | CD45 | PacB | HI30 | biolegend | BSA (Bovine serum Albumin) | | | | Sigma |
| anti-human | CD11c | BV421 | s-hcl-3 | biolegend | Formaldehyde | | | | Fisher |
| anti-human | CD25 | BV421 | M-A251 | biolegend | | | | | |
| anti-human | PD-L1 | BV421 | 29E.2A3 | biolegend | | | | | |
| anti-human | TIM-3 | BV421 | F38-2E2 | biolegend | | | | | |

## Slide 4
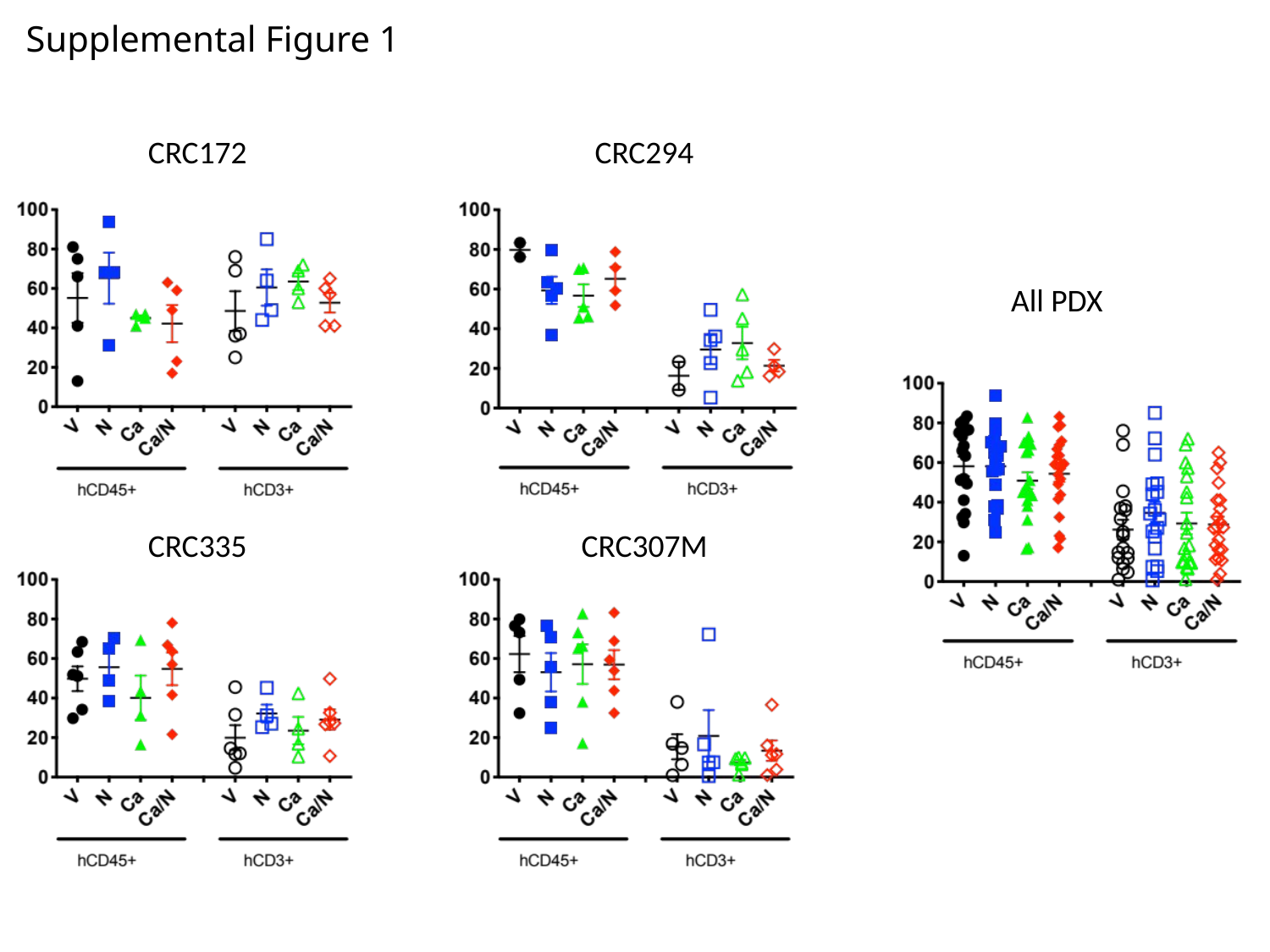

# Supplemental Figure 1
CRC172
CRC294
All PDX
CRC335
CRC307M

## Slide 5
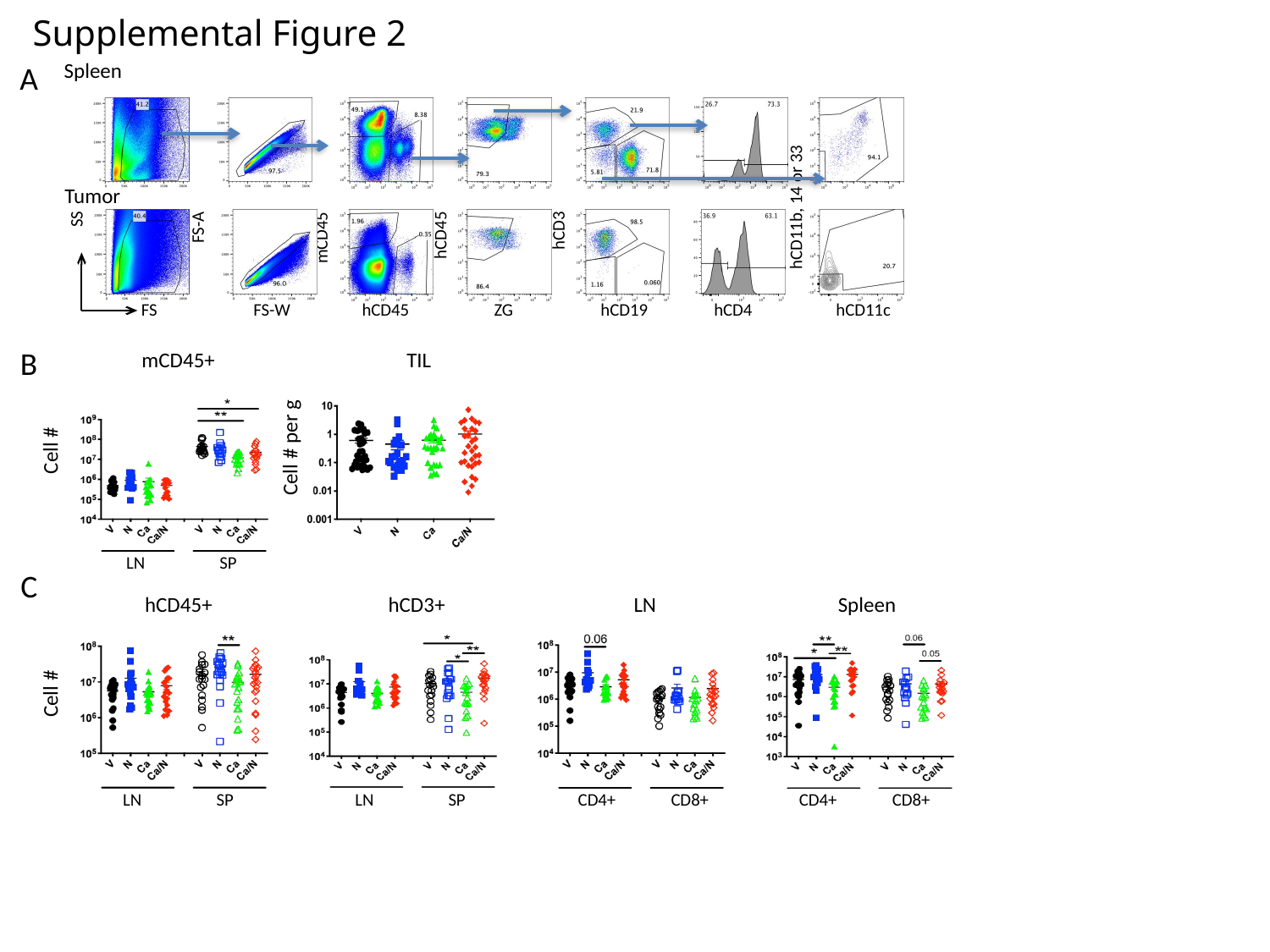

Supplemental Figure 2
Spleen
A
hCD11b, 14 or 33
Tumor
SS
FS-A
mCD45
hCD45
hCD3
FS
FS-W
hCD45
ZG
hCD19
hCD4
hCD11c
B
mCD45+
TIL
Cell # per g
Cell #
LN
SP
C
hCD45+
hCD3+
LN
Spleen
Cell #
LN
SP
LN
SP
CD4+
CD8+
CD4+
CD8+

## Slide 6
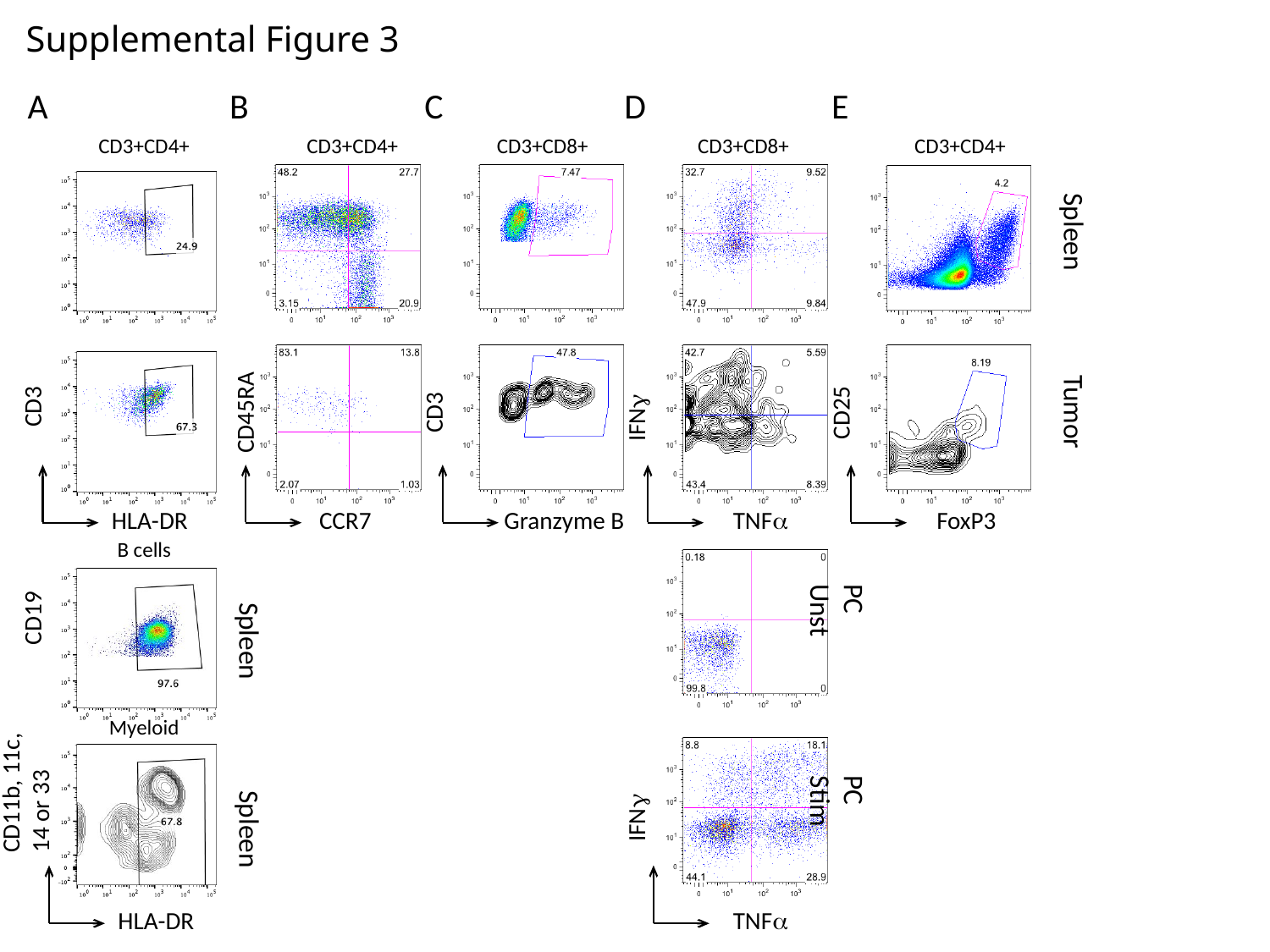

# Supplemental Figure 3
A
B
C
D
E
CD3+CD4+
CD3+CD4+
CD3+CD8+
CD3+CD8+
CD3+CD4+
Spleen
Tumor
CD45RA
CD25
CD3
CD3
IFNg
HLA-DR
CCR7
Granzyme B
TNFa
FoxP3
B cells
PC Unst
Spleen
CD19
CD11b, 11c, 14 or 33
Myeloid
PC Stim
Spleen
IFNg
HLA-DR
TNFa

## Slide 7
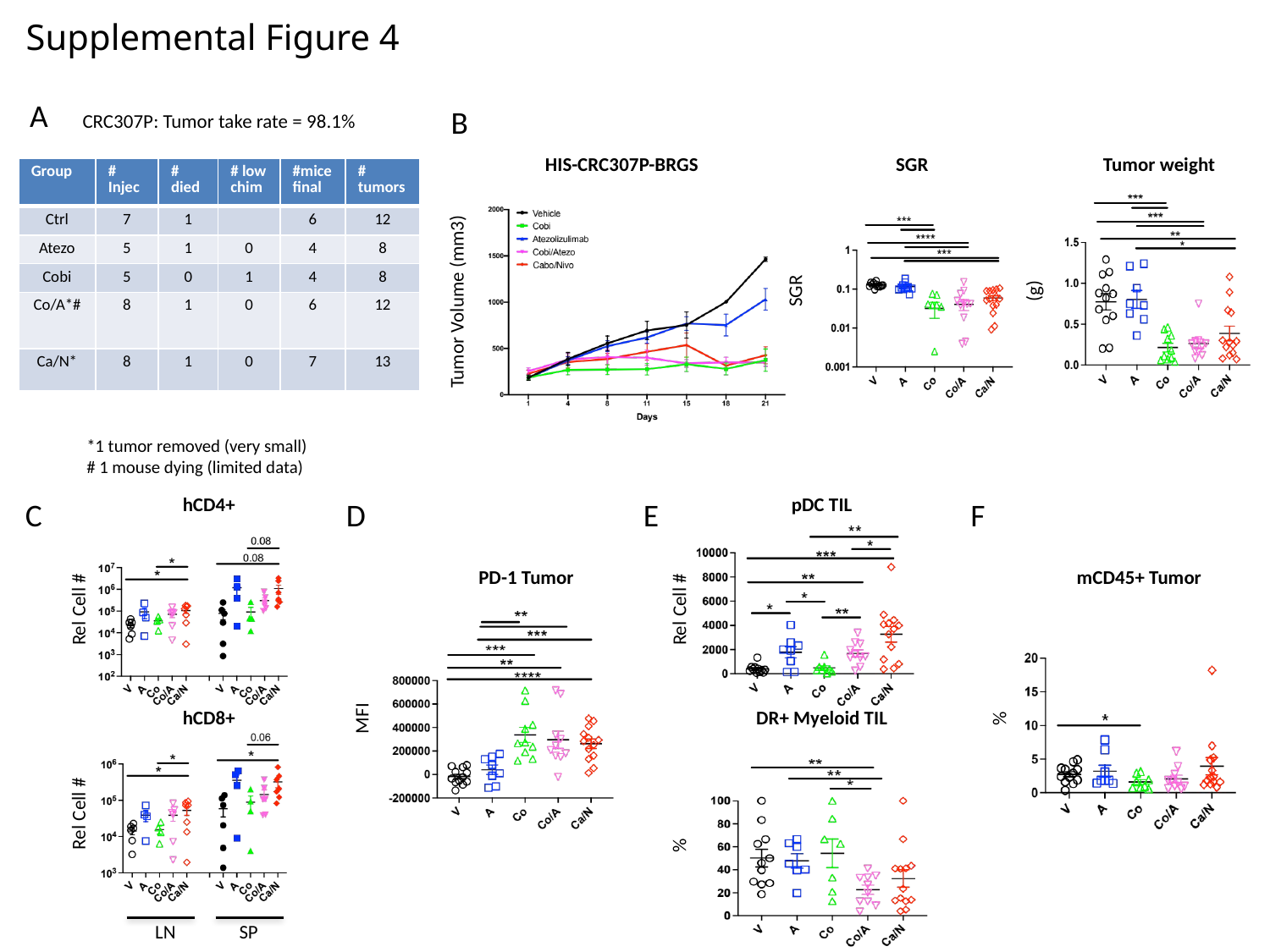

Supplemental Figure 4
A
B
CRC307P: Tumor take rate = 98.1%
HIS-CRC307P-BRGS
SGR
Tumor weight
| Group | # Injec | # died | # low chim | #mice final | # tumors |
| --- | --- | --- | --- | --- | --- |
| Ctrl | 7 | 1 | | 6 | 12 |
| Atezo | 5 | 1 | 0 | 4 | 8 |
| Cobi | 5 | 0 | 1 | 4 | 8 |
| Co/A\*# | 8 | 1 | 0 | 6 | 12 |
| Ca/N\* | 8 | 1 | 0 | 7 | 13 |
Tumor Volume (mm3)
SGR
(g)
*1 tumor removed (very small)
# 1 mouse dying (limited data)
hCD4+
Rel Cell #
hCD8+
LN
SP
Rel Cell #
pDC TIL
Rel Cell #
DR+ Myeloid TIL
%
C
D
E
F
PD-1 Tumor
MFI
mCD45+ Tumor
%

## Slide 8
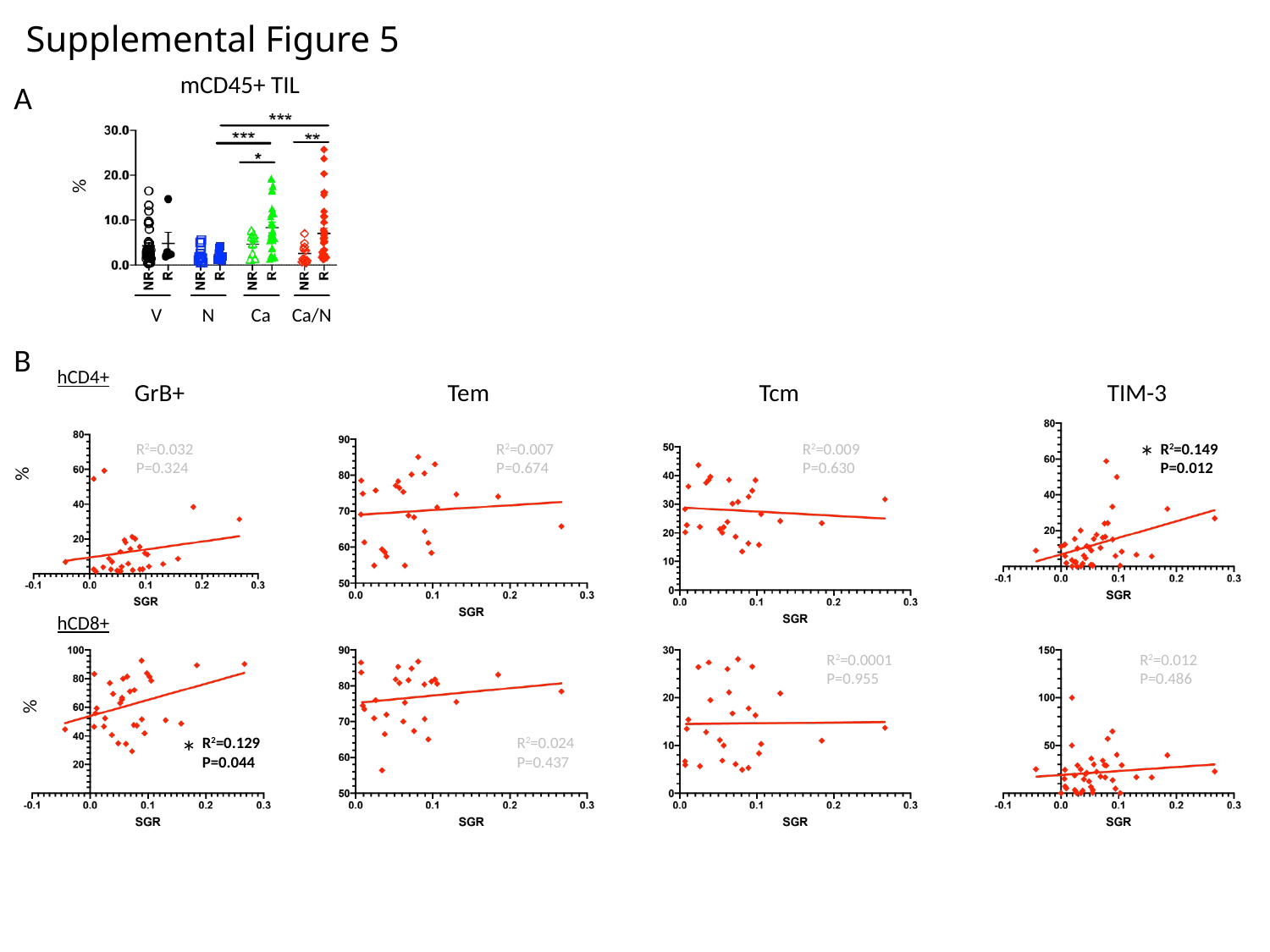

Supplemental Figure 5
mCD45+ TIL
A
%
V
N
Ca
Ca/N
B
hCD4+
GrB+
Tem
Tcm
TIM-3
*
R2=0.032
P=0.324
R2=0.007
P=0.674
R2=0.009
P=0.630
R2=0.149
P=0.012
%
hCD8+
R2=0.0001
P=0.955
R2=0.012
P=0.486
%
*
R2=0.129
P=0.044
R2=0.024
P=0.437

## Slide 9
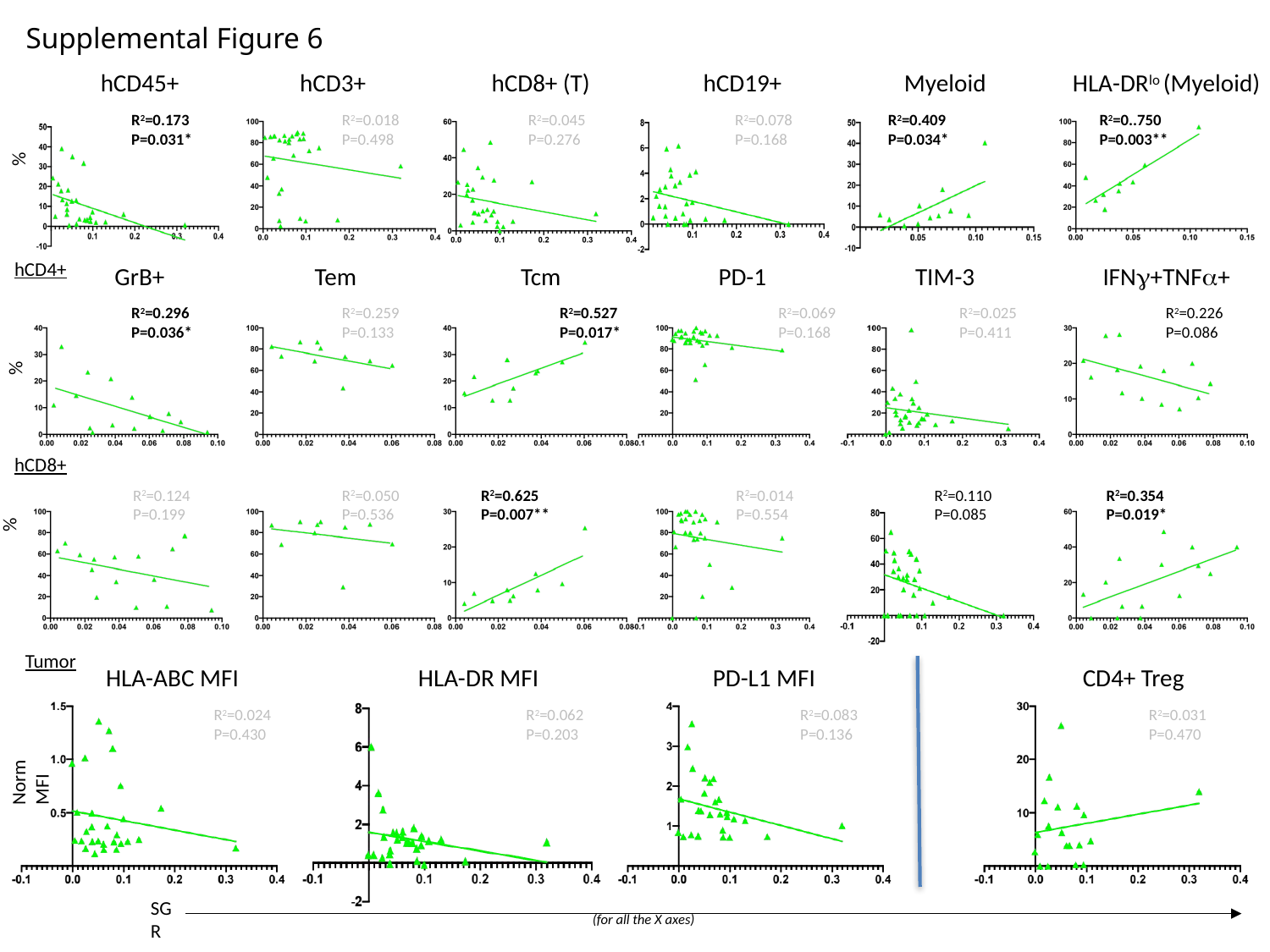

Supplemental Figure 6
hCD45+
hCD3+
hCD8+ (T)
hCD19+
Myeloid
HLA-DRlo (Myeloid)
R2=0.173
P=0.031*
R2=0.018
P=0.498
R2=0.045
P=0.276
R2=0.078
P=0.168
R2=0.409
P=0.034*
R2=0..750
P=0.003**
%
hCD4+
GrB+
Tem
Tcm
PD-1
TIM-3
IFNg+TNFa+
R2=0.296
P=0.036*
R2=0.259
P=0.133
R2=0.527
P=0.017*
R2=0.069
P=0.168
R2=0.025
P=0.411
R2=0.226
P=0.086
%
hCD8+
R2=0.124
P=0.199
R2=0.050
P=0.536
R2=0.625
P=0.007**
R2=0.014
P=0.554
R2=0.110
P=0.085
R2=0.354
P=0.019*
%
Tumor
HLA-ABC MFI
HLA-DR MFI
PD-L1 MFI
CD4+ Treg
R2=0.024
P=0.430
R2=0.062
P=0.203
R2=0.083
P=0.136
R2=0.031
P=0.470
Norm MFI
(for all the X axes)
SGR
